# The Ino80 complex mediates epigenetic centromere propagation via active removal of histone H3

**DOI:** 10.1038/s41467-017-00704-3

**Published:** 2017-09-13

**Authors:** Eun Shik Choi, Youngseo Cheon, Keunsoo Kang, Daeyoup Lee

**Affiliations:** 1 Department of Biological Sciences, Korea Advanced Institute of Science and Technology, Daejeon, 34141 South Korea; 2 Department of Microbiology, Dankook University, Cheonan, Chungnam 31116 South Korea

## Abstract

The centromere is the chromosomal locus at which the kinetochore is assembled to direct chromosome segregation. The histone H3 variant, centromere protein A (CENP-A), is known to epigenetically mark active centromeres, but the mechanism by which CENP-A propagates at the centromere, replacing histone H3, remains poorly understood. Using fission yeast, here we show that the Ino80 adenosine triphosphate (ATP)-dependent chromatin-remodeling complex, which removes histone H3-containing nucleosomes from associated chromatin, promotes CENP-A^Cnp1^ chromatin assembly at the centromere in a redundant manner with another chromatin-remodeling factor Chd1^Hrp1^. CENP-A^Cnp1^ chromatin actively recruits the Ino80 complex to centromeres to elicit eviction of histone H3-containing nucleosomes. Artificial targeting of Ino80 subunits to a non-centromeric DNA sequence placed in a native centromere enhances the spreading of CENP-A^Cnp1^ chromatin into the non-centromeric DNA. Based on these results, we propose that CENP-A^Cnp1^ chromatin employs the Ino80 complex to mediate the replacement of histone H3 with CENP-A^Cnp1^, and thereby reinforces itself.

## Abstract (web summary)

The histone variant CENP-A marks active centromeres and replaces H3 at centromeres through a poorly understood mechanism. Here, the authors provide evidence that the chromatin remodeller Ino80 promotes CENP-A chromatin assembly at the centromere in fission yeast.

## Introduction

In most eukaryotes, centromeres are epigenetically specified by chromatin containing the centromere-specific histone H3 variant, centromere protein A (CENP-A). A model of self-propagating centromeres suggests that pre-established CENP-A chromatin at the centromeres is sufficient to direct replenishment of new CENP-A during the cell cycle^1–5^. At human centromeres, CENP-A replenishment occurs during G1 phase^6^ and is preceded by the replication-independent loss of histone H3.3 incorporated during S phase^7^. This suggests that centromeres possess a mechanism that actively removes histone H3.3 to make room for CENP-A deposition. The fission yeast, *Schizosaccharomyces pombe*, has epigenetically defined “regional centromeres” whose chromatin and protein compositions are similar to those of their human counterparts^8–10^. In fission yeast, CENP-A^Cnp1^ replenishment at centromeres occurs during G2 phase in a DNA replication-independent manner^11^, and histone H3 competes with CENP-A^Cnp1^ for incorporation into centromeres^12^. This suggests that active removal of histone H3 may be a conserved mechanism required for CENP-A deposition at fission yeast centromeres, and makes the fission yeast an attractive genetic model for studying how histone H3 is replaced by CENP-A at regional centromeres.

Adenosine triphosphate (ATP)-dependent chromatin-remodeling factors can disassemble nucleosomes and, in some cases, induce the exchange of histone variants^13–17^. Thus, they are good candidates to be factors through which centromeres induce the removal of histone H3. In support of this possibility, ATP-dependent chromatin-remodeling factors have been implicated in the assembly or maintenance of centromeric chromatin. In *Saccharomyces cerevisiae*, which has genetically defined “point centromeres” consisting of a single CENP-A^Cse4^ nucleosome per centromere, the Ino80 (inositol-requiring mutant 80) ATP-dependent chromatin-remodeling complex acts to maintain normal chromatin structure at centromeres^18^. However, the Ino80 complex in *S. cerevisiae* contributes to the integrity of pericentric chromatin rather than the assembly of CENP-A^Cse4^ nucleosome^18^. In humans, RSF1 (remodeling and spacing factor 1) localizes to centromeres and promotes the stable association of CENP-A with centromeres^19^. A recent study showed that RSF1 can stimulate histone exchange accompanied by CENP-A deposition when artificially targeted to ectopically inserted α-satellite DNA^20^. In addition to RSF1, the conserved ATP-dependent chromatin-remodeling factor, CHD1 (chromodomain helicase DNA binding protein 1), was found to stimulate the deposition of CENP-A^Cnp1^ at centromeres in human and fission yeast^21, 22^. In these studies, however, RSF1 and CHD1 were found to be only partially involved in the deposition of CENP-A at the centromeres. This raises the possibility that other chromatin-remodeling factor(s) are also responsible for regulating CENP-A chromatin assembly at regional centromeres. Also, it remains unclear whether the critical role played by chromatin-remodeling factors at the centromeres is related to the exchange of histone H3 with CENP-A.

To address these questions, here we systematically analyze the roles of the ATP-dependent chromatin-remodeling factors in the centromeric chromatin assembly of fission yeast. We find that the Ino80 complex plays an important role in CENP-A^Cnp1^ chromatin assembly at the fission yeast centromeres in a redundant manner with Chd1^Hrp1^, and the role of the Ino80 complex is linked to removal of histone H3-containing nucleosomes. Consistent with a direct role in CENP-A^Cnp1^ chromatin assembly, the Ino80 complex is associated with centromeric regions in a CENP-A^Cnp1^-dependent manner and when tethered to a non-centromeric DNA inserted in an endogenous centromere promotes spreading of CENP-A^Cnp1^ chromatin into the non-centromeric DNA. Thus, CENP-A^Cnp1^ chromatin utilizes the Ino80 complex together with Chd1^Hrp1^ to mediate replacement of histone H3 with CENP-A^Cnp1^ at regional centromeres of fission yeast.

## Results

### The Ino80 complex promotes CENP-A^Cnp1^ chromatin assembly

**Fig. 1:**
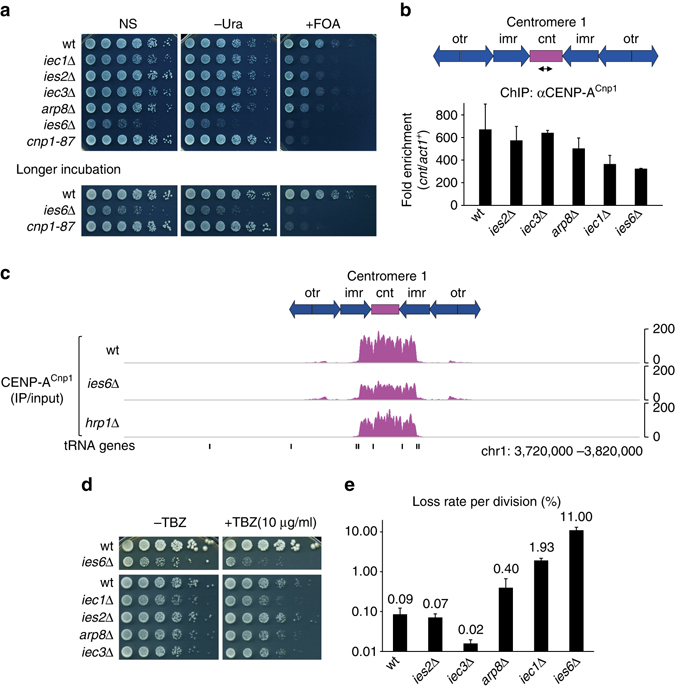
Defects in the Ino80 complex impair assembly and function of centromeric chromatin. **a** Silencing of *ura4* ^+^ inserted at centromere 1 (*cnt1*:*ura4* ^+^) of wild-type (wt) and those harboring mutants of the Ino80 complex. The *ies6*Δ, wt, and CENP-A^Cnp1^ mutant (*cnp1-87*) cells were incubated for one additional day (denoted by “longer incubation”) to ensure full growth. Serial dilutions (fivefold) of cells were spotted onto non-selective (NS), uracil-depleted (−Ura), or FOA-containing (+FOA) medium at 30 °C. FOA was used to kill *ura4* ^+^-expressing cells. **b** Schematic of centromere 1 (*to* *p*), indicating the central core (*cnt*), innermost repeat (*imr*), and outer repeats (*otr/dg-dh*). The primer pairs corresponding to *cnt* are indicated below. ChIP analysis for CENP-A^Cnp1^ in the indicated cells grown at 30 °C (bottom). Fold enrichment was calculated by comparing the *cnt*/*act1* ^*+*^ ratio between the IP and input DNA. Data indicate the mean±s.d. (*error bars*) of three biological replicates. **c** Genome browser view of centromere 1 showing the ChIP-Seq profiles of CENP-A^Cnp1^. Fold enrichment represents IP relative to input. **d** TBZ sensitivity of cells grown with no TBZ (−TBZ) or with 10 μg ml^−1^ TBZ. **e** Minichromosome loss assay monitoring the loss of non-essential chromosome 16 (Ch16) during cell division. Cells (618–4,837) from three independent colonies for each strain were analyzed to estimate the proportion of cells in the colony containing the minichromosome (see Methods for more detail). Data indicate the mean loss rate±s.d. (*error bars*)

To identify the ATP-dependent chromatin-remodeling factors involved in the replacement of histone H3 with CENP-A^Cnp1^ at centromeres, we first examined whether any of the fission yeast chromatin-remodeling factors could affect CENP-A^Cnp1^ chromatin at centromeres. The silencing of *ura4* ^+^ inserted into a central core region of centromere 1 (*cnt1*:*ura4* ^+^) was previously used to assess the integrity of CENP-A^Cnp1^ chromatin in fission yeast^23, 24^. Utilizing this assay, we screened a pool of fission yeast strains carrying mutations in chromatin remodeling factors for defective central core silencing. As previously shown^21, 25^, the loss of Chd1^Hrp1^ disrupted central core silencing (Supplementary Fig. 1a, b). In addition, we found that the loss of Ies2, a non-essential component of the Ino80 chromatin-remodeling complex, also disrupted silencing (Supplementary Fig. 1b). Other viable mutants of the Ino80 complex, including *iec1*Δ and *ies6*Δ, were also defective in silencing (Fig. 1a). Consistent with these findings, chromatin immunoprecipitation (ChIP) analyses showed that mutations of the Ino80 complex, most notably *iec1*Δ and *ies6*Δ, reduced the occupancy of CENP-A^Cnp1^ in the central core region (Fig. 1b and Supplementary Fig. 1c). Using ChIP combined with high-throughput sequencing analysis (ChIP-Seq), we observed that the CENP-A^Cnp1^ levels at all centromeres were majorly reduced in *ies6*Δ cells and to a smaller degree in *hrp1*Δ cells (Fig. 1c and Supplementary Fig. 2). Defects in centromere function often increase the sensitivity of cells to microtubule-destabilizing drugs, such as thiabendazole (TBZ), and increase the rates of chromosome loss^24, 26^. We found that *iec1*Δ and *ies6*Δ induce TBZ sensitivity and dramatically increase the loss of a non-essential minichromosome (Fig. 1d, e), indicating that the impaired assembly of CENP-A^Cnp1^ chromatin in cells harboring defective Ino80 complex can disrupt normal centromere function.

### The Ino80 complex evicts histone H3 from chromatin

**Fig. 2:**
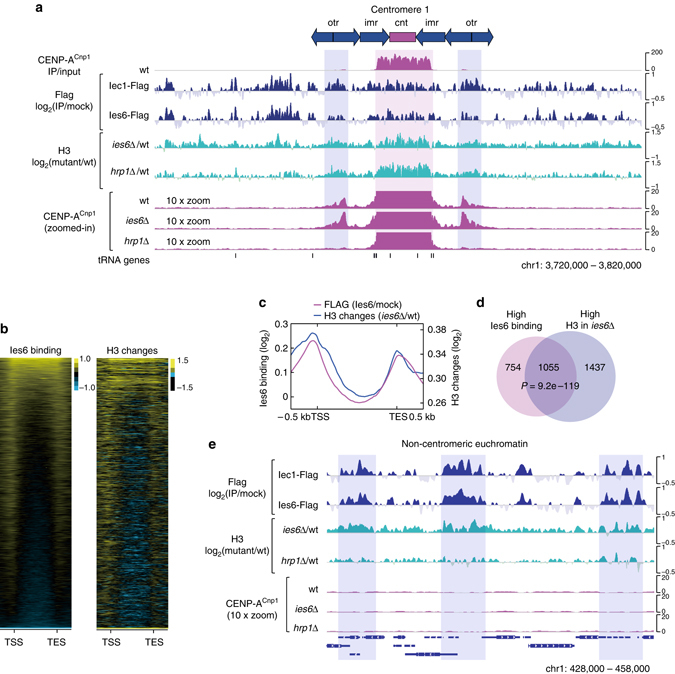
The Ino80 complex induces removal of histone H3 from associated chromatin. **a** ChIP-Seq profiles of CENP-A^Cnp1^, Iec1-5×Flag, and Ies6-5×Flag in wt cells, and the histone H3 changes in *ies6*Δ and *hrp1*Δ cells relative to wt at centromere 1. The ChIP enrichments for Flag-tagged Ino80 subunits were normalized to those in mock (no tag) cells. The H3 ChIP-Seq experiments were performed and analyzed using wild-type *S. cerevisiae* cells as external spike-in controls (see Methods for more detail). Magnified (×10) views of CENP-A^Cnp1^ are shown below. The *magenta* and *blue vertical shadings* indicate the major CENP-A^Cnp1^ peaks in the central domain and the minor CENP-A^Cnp1^ peaks in the heterochromatin, respectively. **b** Heatmaps representing Ies6 binding in wt cells (*left*) and H3 occupancy changes in *ies6*Δ cells (*right*) across all protein-coding and non-coding genes of *S. pombe* (from −0.5 kb of the TSS to +0.5 kb of the TES; *n* = 7,106). The heatmaps were sorted in descending order of Ies6 enrichment. TES, transcription termination site; TSS, transcription start site. **c** Average gene profiles (*n* = 7,106) for Ies6 binding and histone H3 changes in *ies6*Δ cells. **d** Venn diagram showing overlap of genes with high Ies6 binding and those with high histone H3 increases in *ies6*Δ cells, as individually determined by *k*-means clustering (*k* = 2). *P*-values were calculated using the hypergeometric distribution. **e** ChIP-Seq profiles at a representative euchromatic region for: Iec1-5×Flag; Ies6-5×Flag; histone H3 changes in *ies6*Δ and *hrp1*Δ cells relative to wt; and CENP-A^Cnp1^ in wt, *ies6*Δ, and *hrp1*Δ cells

Next, we used ChIP-Seq to determine whether the Ino80 complex acts directly at centromeres. This analysis revealed that the Iec1 and Ies6 subunits were widely associated with central kinetochore domains (i.e., *cnt* and the inner parts of *imr*), as well as with specific regions of pericentromeric heterochromatin (*otr*) and proximal euchromatin (Fig. 2a and Supplementary Fig. 3). Notably, we found that localization of the Ino80 subunits in these regions coincided with the increased levels of histone H3 in *ies6*Δ and *hrp1*Δ cells (Fig. 2a and Supplementary Fig. 3), directly implicating the Ino80 complex and Chd1^Hrp1^ in the removal of histone H3-containing nucleosomes^27^. We observed that low levels of CENP-A^Cnp1^ incorporation were frequently observed at or near the centromere-proximal regions (pericentromeric heterochromatin and proximal euchromatin) where the Ino80 complex and Chd1^Hrp1^ act to remove histone H3 (Fig. 2a and Supplementary Fig. 3). This suggests that the active removal of histone H3-containing nucleosomes by chromatin-remodeling factors predisposes these regions to incorporate CENP-A^Cnp1^ nucleosomes. The weak enrichment of CENP-A^Cnp1^ at these centromere-proximal regions was impaired in *hrp1*Δ cells, but not in *ies6*Δ cells (Fig. 2a and Supplementary Fig. 3). This indicates that the Ino80 complex acts primarily to remove histone H3-containing nucleosomes, which leads to the assembly of CENP-A^Cnp1^ in the central domain. However, unlike Chd1^Hrp1^, the Ino80 complex outside the central domain may guide only futile nucleosome exchanges, such as the reciprocal exchange of a histone H3-containing nucleosome with a CENP-A^Cnp1^ nucleosome, or the exchange of one histone H3-containing nucleosome with another. In contrast to *ies6*Δ, the *mis18-262* mutation, which disrupts kinetochore assembly^28^, was associated with reduced CENP-A^Cnp1^ nucleosome assembly at both central domains and centromere-proximal regions (Supplementary Fig. 4). This suggests that the Ino80 complex is not involved in the kinetochore-dependent deposition or maintenance of CENP-A^Cnp1^. To test this further, we analyzed the effect of *ies6*Δ on the centromeric localization of Scm3, a CENP-A^Cnp1^-specific histone chaperone orthologous to human HJURP^29–33^. The association of Scm3 with centromeres is known to require various kinetochore proteins, including Sim4, Mis6, Mis16, and Mis18, but not CENP-A^Cnp1^ itself^[ 29^. We found that binding of Scm3 to the centromere is not significantly impaired in *ies6*Δ cells (Supplementary Fig. 5), indicating that the CENP-A^Cnp1^ deposition pathway is likely to be intact in *ies6*Δ cells.

As in budding yeast, the Ino80 subunits of fission yeast are also associated with other genomic loci, especially near the start and end of genes^34, 35^ (Fig. 2b, c and Supplementary Fig. 6a, b). This non-centromeric localization of the Ino80 complex allowed us to examine whether the Ino80 complex acts to remove histone H3-containing nucleosomes regardless of CENP-A^Cnp1^ chromatin assembly. Indeed, and consistent with the notion that the Ino80 complex plays a direct role in histone H3 removal^27, 36^, we found that the levels of histone H3 in *ies6*Δ cells were increased in regions that are normally bound by Ies6 (Fig. 2b, c). Corroborating this, a Venn diagram analysis revealed that there is a significant overlap between genes showing high levels of Ies6 binding and those exhibiting robust increases in H3 levels in *ies6*Δ cells (Fig. 2d). We found that Chd1^Hrp1^ also promotes histone H3 removal at non-centromeric regions similar to those targeted by the Ino80 complex (near the start and end of genes), but it plays a weaker role in evicting H3 from euchromatin (Fig. 2e and Supplementary Fig. 7a, b). We also confirmed that the removal of histone H3 by the Ino80 complex at euchromatin is not tied to the assembly of CENP-A^Cnp1^ (Fig. 2e and Supplementary Fig. 7c).

In budding yeast, the Ino80 complex acts to remove the histone H2A variant, H2A.Z^Htz1^, from chromatin^16, 34^. Since H2A.Z^Pht1^ is depleted from fission yeast centromeres^37, 38^, we questioned whether the defective CENP-A^Cnp1^ chromatin assembly of *ies6*Δ cells could be caused by the insufficient removal of H2A.Z^Pht1^, rather than H3, from the centromeres. In sharp contrast to the prominent increase in H3 occupancy seen in *ies6*Δ cells, however, we found that *ies6*Δ does not cause a significant increase in H2A.Z^Pht1^ occupancy at the centromeres (Supplementary Fig. 8a, b). Moreover, loss of H2A.Z^Pht1^ did not rescue the defective central core silencing of *ies2*Δ cells (Supplementary Fig. 8c), indicating that this silencing defect is not caused by H2A.Z^Pht1^. As previously reported in budding yeast^16, 34^, we observed that H2A.Z^Pht1^ accumulates at both gene promoters and coding regions in *ies6*Δ cells (Supplementary Fig. 8d, e). We thus conclude that the Ino80 complex affects CENP-A^Cnp1^ chromatin in a manner independent of its role in restricting the localization of H2A.Z^Pht1^.

The action of the Ino80 complex at gene promoters prompted us to question whether the observed effect of *ies6*Δ on CENP-A^Cnp1^ chromatin could be indirectly caused by alterations in the expression levels of specific factors that regulate CENP-A^Cnp1^ chromatin assembly. To exclude this possibility, we used mRNA-Seq analysis to perform gene expression profiling of *ies6*Δ and wild-type (wt) cells. Consistent with the notion that the Ino80 complex plays a role in transcription, we identified hundreds of genes whose mRNA levels are significantly down- or up-regulated in *ies6*Δ cells (Supplementary Table 1). However, among the genes whose functions are known to be important for CENP-A^Cnp1^ chromatin assembly, only *cnp3* ^+^, which encodes the ortholog of the CENP-A^Cnp1^-binding protein, CENP-C^Cnp3^, in *S. pombe* ^39^, showed significantly reduced expression in *ies6*Δ cells (Supplementary Table 1). A noticeable observation that we made to solve this issue was that under a suboptimal growth condition (at 25 °C in minimal EMM), *ies6*Δ does not downregulate *cnp3* ^+^ expression (Supplementary Fig. 9a), but *ies6*Δ cells grown under this growth condition still show a significantly reduced level of CENP-A^Cnp1^ at the centromere (Supplementary Fig. 9b). Therefore, we conclude that the Ino80 complex regulates CENP-A^Cnp1^ chromatin regardless of its involvement in regulating *cnp3* ^+^ expression.

### The Ino80 complex functions redundantly with Chd1^Hrp1^

The weaker effect of *hrp1*Δ on CENP-A^Cnp1^ chromatin suggests that Chd1^Hrp1^ may complement the function of the Ino80 complex. We were unable to generate *ies6*Δ *hrp1*Δ cells, suggesting that *ies6*Δ and *hrp1*Δ are synthetically lethal. The *ies2*Δ *hrp1*Δ double-mutant cells were viable, and showed larger defects in central core silencing than those observed in each of the single mutants (Supplementary Fig. 10a). The combination of *hrp1*Δ and a mutation affecting the RSC (remodeling the structure of chromatin) chromatin-remodeling complex^40^ did not produce this additive defect in silencing (Supplementary Fig. 10b, c), nor did the RSC mutant (*rsc1*Δ) cause any silencing defect when combined with *ies2*Δ (Supplementary Fig. 10d). To further investigate the functional redundancy of Ino80 and Chd1^Hrp1^, we generated strains carrying mutations in the essential catalytic subunit, Ino80, which show a cold-sensitive central core silencing defect (see Methods for more detail). When we combined the silencing-defective *ino80-11* with *hrp1*Δ, we observed a cumulative loss of both silencing and cell viability (Supplementary Fig. 11a). Consistent with this, we found that the combination of *ino80-11* with *hrp1*Δ produces a more prominent loss of CENP-A^Cnp1^ at the centromere than either of the single mutations (Supplementary Fig. 11b). The level of histone H3 occupancy at the centromere was also increased most significantly in the double mutant (*ino80-11 hrp1*Δ) compared to the wt (Supplementary Fig. 11c). These data indicate that Ino80 and Chd1^Hrp1^ share a role in promoting the replacement of histone H3 with CENP-A^Cnp1^.

### CENP-A^Cnp1^ chromatin actively recruits the Ino80 complex

**Fig. 3:**
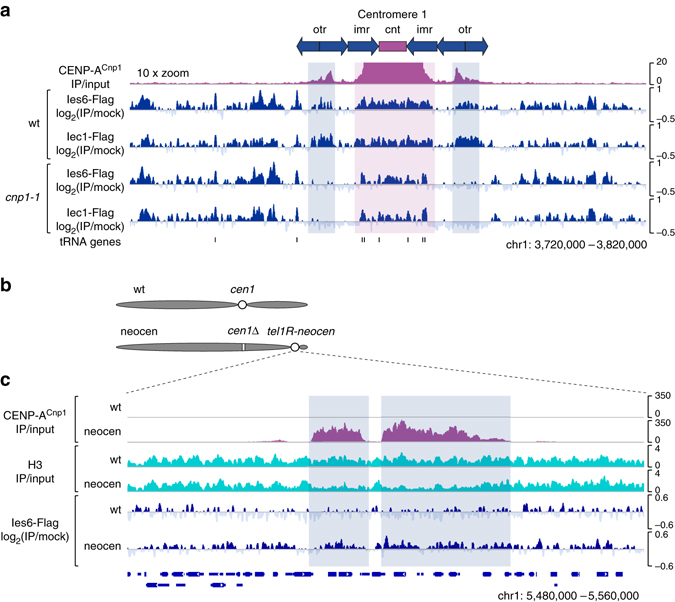
The Ino80 complex is recruited to centromeres through CENP-A^Cnp1^chromatin. **a** Magnified (×10) views of CENP-A^Cnp1^ in wt cells and ChIP-Seq profiles of Ies6-5×Flag and Iec1-5×Flag in wt and *cnp1-1* cells at centromere 1. For Flag ChIP-Seq, wt and *cnp1-1* cells were incubated at 36 °C (restrictive temperature for *cnp1-1*) for 3 h after being grown at 25 °C (permissive temperature for *cnp1-1*). The *magenta* and *blue vertical shadings* indicate the major CENP-A^Cnp1^ peaks in the central domain and the minor CENP-A^Cnp1^ peaks in the heterochromatin, respectively. **b** A schematic showing the positions of active centromeres (*white circles*) in chromosome 1 of the wt and neocentromere (neocen) strains. The neocentromere strain carries a neocentromere (*tel1R-neocen*) assembled at a subtelomeric region, but lacks the endogenous centromere 1 (*cen1*Δ; *white box*). **c** Genome browser view of the *tel1R-neocen* locus showing the ChIP-Seq profiles of CENP-A^Cnp1^, histone H3, and Ies6-5×Flag in the wt and neocentromere strains. The *blue vertical shadings* indicate the major CENP-A^Cnp1^ peaks in the neocentromere

The association of the Ino80 complex with specific non-centromeric regions, such as gene promoters, prompted us to ask whether the centromeric localization of the Ino80 complex is mediated by CENP-A^Cnp1^ chromatin or by specific features of centromeric DNA sequences. To address this question, we examined whether CENP-A^Cnp1^ is required for the centromeric localization of the Ino80 complex. We found that in cells with defective CENP-A^Cnp1^ (*cnp1-1*), the overall levels of Iec1 and Ies6 bound to the central domain (*magenta vertical shading*) were diminished and became constrained to more specific regions, including the transfer RNA genes (Fig. 3a). Interestingly, the association of the Ino80 complex with pericentromeric heterochromatin regions (*otr*) harboring low levels of CENP-A^Cnp1^ (*blue vertical shading*) was completely abolished in *cnp1-1* cells (Fig. 3a). These results indicate that the widespread associations of the Ino80 complex with CENP-A^Cnp1^-enriched domains occur through CENP-A^Cnp1^. However, we cannot rule out the possibility that DNA sequence features may also play some role^41^. We observed that CENP-A^Cnp1^, when overexpressed, co-immunoprecipitates with Ies6, which raises the possibility that CENP-A^Cnp1^ may have an interaction with the Ino80 complex (Supplementary Fig. 12). To further evaluate the significance of CENP-A^Cnp1^ chromatin or DNA sequence cues in the centromeric localization of the Ino80 complex, we utilized a neocentromere strain that lacks the endogenous centromere of chromosome 1 and carries an ectopic centromere assembled at a non-centromeric region near the right telomere of chromosome 1 (*tel1R-neocen*) without any DNA sequence change^42^ (Fig. 3b). We found that Ies6 is widely associated with the *tel1R-neocen* locus in the neocentromere strain but not in the wt strain (Fig. 3c). Thus, CENP-A^Cnp1^ chromatin, not the DNA sequence, majorly contributes to localizing the Ino80 complex to the neocentromere.

### Tethered Ino80 subunits promote spreading of CENP-A^Cnp1^

**Fig. 4:**
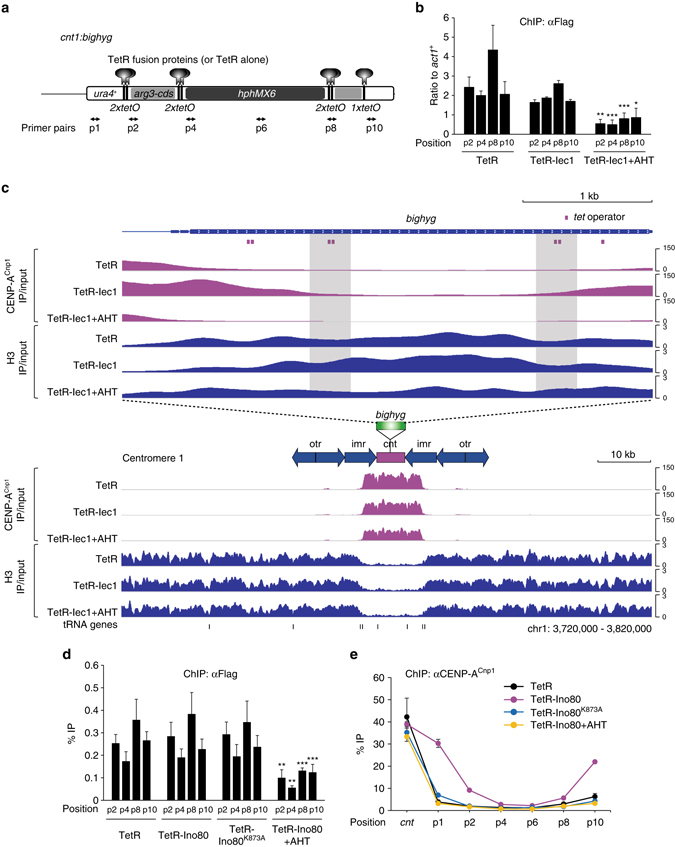
Tethered Ino80 subunits enhance spreading of CENP-A^Cnp1^ chromatin into a non-centromeric DNA. **a** A schematic of *cnt1*:*bighyg* showing the positions of the *tet* operators (*tetO*), the TetR fusion proteins (or TetR alone) in *bighyg*. The *bighyg* DNA consists of *ura4* ^+^, *arg3* ^+^ coding sequence (*arg3-cds*), and the hygromycin-resistance gene (*hphMX6*). The utilized primers pairs are indicated below. **b** ChIP analysis for Flag-tagged TetR, TetR-Iec1, and TetR-Iec1 in cells treated with AHT (TetR-Iec1+AHT), as assessed using the indicated primers. TetR-Iec1 binding was significantly reduced after the addition of AHT, as determined by Student’s *t*-tests (*P ≤ 0.05, **P ≤ 0.01, and ***P ≤ 0.001). **c** Genome browser view of *bighyg* (*top*) and centromere 1 (*bottom*) showing the ChIP-Seq profiles of CENP-A^Cnp1^ (*magenta*) and histone H3 (*blue*) in TetR-expressing cells, TetR-Iec1-expressing cells, and TetR-Iec1-expressing cells treated with AHT (TetR-Iec1+AHT). *Gray vertical shadings* indicate ~400 bp regions encompassing *tetO* inserted in the central part of *bighyg*. **d** ChIP analysis for Flag-tagged TetR, TetR-Ino80, TetR-Ino80^K873A^, and TetR-Ino80 in cells treated with AHT (TetR-Ino80+AHT), as assessed using the indicated primers. **e** ChIP analysis for CENP-A^Cnp1^ in cells expressing Flag-tagged TetR, TetR-Ino80, and TetR-Ino80^K873A^, and in TetR-Ino80-expressing cells treated with AHT (TetR-Ino80+AHT), as assessed using the indicated primers. *Error bars* in all ChIP data indicate the ±s.d. for the biological replicates

A large non-centromeric DNA placed in an endogenous centromere was previously shown to act as a poor substrate for the spreading of CENP-A^Cnp1^ chromatin^43^. We used this to more directly address the function of the Ino80 complex in CENP-A^Cnp1^ chromatin assembly. We tethered a TetR-2×Flag-Iec1 (TetR-Iec1) fusion to *tetO* sites in a 4.5 kb non-centromeric DNA (*bighyg*) sequence inserted at centromere 1, and examined whether tethered TetR-Iec1 alters CENP-A^Cnp1^ chromatin assembly at *bighyg* (Fig. 4a). We used TetR-2×Flag alone (TetR) and TetR-Iec1 released from *bighyg* by anhydrotetracycline (AHT) treatment as negative controls. We used ChIP analysis to confirm that TetR and TetR-Iec1 were enriched at *tetO* insertions and that TetR-Iec1 was released after the addition of AHT (Fig. 4b). A subsequent ChIP-Seq analysis showed that tethered TetR-Iec1 triggered the spread of CENP-A^Cnp1^ chromatin into *bighyg* concurrent with a decline in histone H3 occupancy (Fig. 4c, compare TetR-Iec1 with TetR or TetR-Iec1+AHT). Tethering of TetR-Iec1 to more central regions of *bighyg* did not cause such a robust increase in CENP-A^Cnp1^ chromatin assembly (*gray vertical shadings* in Fig. 4c). However, TetR-Iec1 tethered to these regions causes a more notable local histone H3 depletion relative to surrounding regions than did tethered TetR (*gray vertical shadings* in Fig. 4c), which is consistent with the notion that TetR-Iec1 induces the removal of histone H3-containing nucleosomes around the tethered sites. To determine whether the effect of tethered TetR-Iec1 reflects the underlying catalytic activity of the Ino80 complex, we ectopically expressed TetR-Ino80 or TetR-Ino80^K873A^, which has a mutation in its conserved ATP-binding pocket^44, 45^. We observed similar enrichments for TetR, TetR-Ino80, and TetR-Ino80^K873A^ at *tetO* insertions, and TetR-Ino80 was successfully released after the addition of AHT (Fig. 4d). However, tethered TetR-Ino80, but not TetR-Ino80^K873A^, caused the spreading of CENP-A^Cnp1^ chromatin into *bighyg* (Fig. 4e, compare TetR-Ino80 and TetR-Ino80^K873A^ at p1, p2, and p10), clearly implicating the ATPase activity of Ino80 in CENP-A^Cnp1^ chromatin assembly. Moreover, we found that tethered Ino80 subunits exert a stronger effect on CENP-A^Cnp1^ assembly when placed closer to the native centromere (Fig. 4c–e). This supports that the Ino80 complex needs to be in a kinetochore context to stimulate CENP-A^Cnp1^ chromatin assembly, which is consistent with the observation that CENP-A^Cnp1^ does not accumulate at non-centromeric regions where the Ino80 complex acts to remove histone H3 (Fig. 2e and Supplementary Fig. 7c).

## Discussion

This study shows that the Ino80 complex is a critical chromatin-remodeling factor required for CENP-A^Cnp1^ chromatin assembly at the centromeres of fission yeast. Chd1^Hrp1^, which was previously implicated in CENP-A^Cnp1^ chromatin assembly^21^, plays a redundant role with the Ino80 complex. Our findings in fission yeast may explain the discrepancy between human and *Drosophila* regarding the involvement of CHD1 in CENP-A chromatin assembly^22, 46^, as the function of CHD1 could be easily concealed by the redundant function of other chromatin-remodeling factors such as the Ino80 complex.

Our conclusion that CENP-A^Cnp1^ chromatin rather than specific DNA sequence is the major determinant for centromeric localization of the Ino80 complex is supported by two main observations. First, the binding of Ino80 subunits is detected across the CENP-A^Cnp1^ chromatin domains, and this widespread association is largely dependent on CENP-A^Cnp1^ (Fig. 3a). Second, Ies6 associates with the site of neocentromere formation only when the neocentromere is activated and CENP-A^Cnp1^ chromatin is assembled on the DNA (Fig. 3c).

Previous studies showed that the Ino80 complex in budding yeast also associates with the centromeres^47^ and promotes pericentric chromatin integrity and centromere function^18^, but does not affect the assembly of CENP-A^Cse4^ nucleosomes at the centromeres^18^. A recent study uncovered a rather unexpected role for the budding yeast Ino80 complex in promoting the ectopic deposition of CENP-A^Cse4^ at gene promoters^48^. We speculate that the requirement for the Ino80 complex in the assembly of CENP-A nucleosomes at the centromeres was lost in budding yeast during the evolutionary transition from regional to point centromeres^49^. In contrast, the connection of the Ino80 complex with centromeres^18^ and CENP-A^48^ was retained, allowing the Ino80 complex to exhibit divergent roles in budding yeast. The roles of the Ino80 complex in CENP-A chromatin assembly at regional versus point centromeres should be examined in the future to test this hypothesis.

As reported in budding yeast^16, 34^, we herein show that the fission yeast Ino80 complex plays a prominent role in removing histone H2A.Z^Pht1^ from chromatin (Supplementary Fig. 8d, e). However, we found that H2A.Z^Pht1^ does not significantly accumulate at the centromeres in *ies6*Δ cells (Supplementary Fig. 8a, b) and the defective central core silencing in *ies2*Δ cells is not rescued by the elimination of histone H2A.Z^Pht1^ (Supplementary Fig. 8c). These results indicate that the major function of the Ino80 complex in the assembly of CENP-A^Cnp1^ chromatin at the centromeres is not likely to be the removal of histone H2A.Z^Pht1^. Instead, our analysis revealed that the localization of the Ino80 complex was tightly correlated with its role in histone H3 eviction rather than CENP-A^Cnp1^ deposition (Fig. 2). Our findings thus support a model in which the Ino80 complex mainly acts at the centromeres to remove histone H3-containing nucleosomes as a prerequisite for CENP-A^Cnp1^ deposition. We note that the Ino80 complex was also shown to induce full nucleosome turnover in budding yeast^34^. Kinetochore proteins, such as CENP-C^Cnp3^, may stabilize CENP-A^Cnp1^ nucleosomes at centromeres^50^, thereby generating a chromatin context in which the Ino80 complex selectively evicts histone H3-containing nucleosomes. Extending our model, a recent study showed that the chromatin-remodeling factor, RSF1, can stimulate histone H3.3 exchange as well as CENP-A deposition in humans^20^. Based on the present and prior findings, we envision that the exchange of histone H3-containing nucleosomes with CENP-A nucleosomes by centromere-targeted chromatin-remodeling factors could be a conserved theme underpinning the epigenetic propagation of CENP-A chromatin at regional centromeres.

## Methods

### Strains, drugs and standard techniques

Standard genetic and molecular techniques were used. YES (Yeast Extract with Supplements) was used as a rich medium and PMG (Pombe Minimal Glutamate) or EMM (Edinburgh Minimal Medium) was used as a minimal medium. For the selective and non-selective media in the silencing assays, we used PMG supplemented with amino acids. For 5-fluoroorotic acid (FOA) selection, we added FOA (final concentration 1 g l^−1^) to PMG media. Where indicated, we added TBZ (final concentration 10 μg ml^−1^; Sigma) dissolved in dimethyl sulfoxide to the YES plates and AHT (final concentration 10 μM; Sigma) dissolved in DMSO to the YES or PMG media. We incubated all fission yeast cells at 30 °C unless otherwise indicated. We used PCR-based gene targeting or genetic crosses followed by random spore analysis to create the deletion and Flag-tagged strains. We were able to infer the synthetic lethality of *ies6*Δ *hrp1*Δ by crossing *ies6*Δ and *hrp1*Δ and performing a random spore analysis; we were unable to find any *ies6*Δ::*natMX6 hrp1*Δ::*kanMX6* (*nat* ^R^ *kan* ^R^) spores despite selecting over 300 *ies6*Δ::*natMX6* (*nat* ^R^) spores. For more details regarding the yeast strains used in each experiment, see Supplementary Table 2.

### ChIP-Seq and data analysis

Cells were fixed with 1% formaldehyde for 10 min (for cells grown at 36 °C), 15 min (for cells grown at 30 °C), or 40 min (for cells grown at 20 °C). ChIP-Seq required more cells than conventional ChIP, as we needed to ensure that there was sufficient immunoprecipitated DNA for high-throughput sequencing. We used 1.6 × 10^9^, 4 × 10^8^, and 2.4–3.2 × 10^9^ cells for the CENP-A^Cnp1^ChIP, H3 ChIP, and Flag ChIP experiments, respectively, increasing the amount of lysis buffers and antibodies accordingly. For the CENP-A^Cnp1^and H3 ChIP-Seq, we used 70 µl of in-house rabbit anti-CENP-A^Cnp1^ antiserum and 15 µl of in-house rabbit anti-H3 antiserum, respectively. For the Flag ChIP-Seq experiments, we used 10 µl of anti-Flag M2 antibody (1 mg ml^−1^, mouse monoclonal, F1804; Sigma) and included equal numbers of wt cells (lacking any tag) in parallel as controls. We performed the ChIP experiments for CENP-A^Cnp1^ and H3 with only minor modifications to conventional ChIP protocols. We prepared the cell extracts by disrupting the cells in lysis buffer (50 mM HEPES-KOH, pH 7.5, 140 mM NaCl, 1 mM EDTA, 1% Triton X-100, 0.1% sodium deoxycholate) using a standard bead-beating protocol. Following immunoprecipitation (IP), we washed the DNA-bound protein Aagarose beads and eluted the DNA in IP columns (SigmaPrep spin columns, Sigma) instead of microfuge tubes. As the external spike-in control for the H3 ChIP-Seq experiments, formaldehyde-fixed wild-type *S. cerevisiae* (W303a) cells were added to the *S. pombe* cells at a 1:8 ratio before cell disruption. For the Flag ChIP experiments, we used a modified lysis buffer (50 mM Tris-HCl, pH 7.5, 150 mM NaCl, 1 mM EDTA, 1% Triton X-100) and Tris-buffered saline (TBS) (20 mM Tris-HCl, pH 7.5, 150 mM NaCl)-based wash buffers (TBS and TBS with 0.05% Tween-20) to avoid denaturation of the M2 antibody by sodium deoxycholate. As the external spike-in control for the H2A.Z^Pht1^-5×Flag ChIP-Seq experiments, formaldehyde-fixed *S. cerevisiae* cells expressing Sua7-5×Flag were added to *S. pombe* cells at a 1:9 ratio before cell disruption. We recovered the de-crosslinked DNA using a Qiagen PCR purification kit. We constructed the ChIP-Seq libraries with 5 to 10 ng of input DNA or 1 to 10 ng of immunoprecipitated DNA, using a ChIP-Seq kit (NEXTflexChIP-Seq kit, Bioo Scientific) according to the manufacturer’s protocol. For multiplexed libraries, we sequenced 50 nt single-end reads on an Illumina HiSeq 2500 (Theragen and Macrogen). After removing the adapter sequences, we aligned the processed reads to the *S. pombe* ASM294v2 genome assembly using Novoalign. We randomly assigned single locations to reads that mapped to multiple locations. For reads obtained from duplicate samples, we confirmed their experimental reproducibility using the bamCorrelate tool from the deepTools package^51^. For quantitative analysis of the data obtained from the H3 and H2A.Z^Pht1^ChIP-Seq experiments, we calculated the IP/input ratio for each ChIP normalized to that of the spike-in control, which in principle should be constant in all ChIP samples. The normalized ratio of IP/input (E^IP^) was derived as:

E^IP^=(read count of IP mapped to *S. pombe* genome/read count of input mapped to *S. pombe* genome)/(read count of IP mapped to *S. cerevisiae* genome/read count of input mapped to *S. cerevisiae* genome).

We computed the fold change of IP versus the input using the deepToolsbamCompare tool. The normalization factor (*N*) for the IP/input in each ChIP-Seq experiment was calculated as:

*N*×read count of IP/read count of input=E^IP^:1.

When we directly computed the fold change of IP in the mutant versus wild type, the normalization factor (*N*) for IP^mutant^/IP^wt^ was calculated as:

*N*×read count of IP^mutant^/read count of IP^wt^=E^IP^ of mutant/E^IP^ of wt.

The normalization of ChIP-Seq for CENP-A^Cnp1^, Iec1-5×Flag and Ies6-5×Flag was performed using the signal extraction scaling method. For visualization of the normalized data, we used the integrative genome viewer. We used the deepTools computeMatrix tool to perform an average gene (metagene) analysis and the profiler tool from deepTools to visualize the average gene profiles. We generated heatmaps using the deepToolsHeatmapper. We performed all further downstream bioinformatic analyses of the ChIP-Seq data using the R software package.

### ChIP analysis

We performed the Fast ChIP protocol^52^ using 20 µl of anti-CENP-A^Cnp1^ antibody (rabbit polyclonal; in-house), 8 µl of anti-H3 antibody (rabbit polyclonal; in-house), 2 µl of anti-Flag M2 antibody (1 mg ml^−1^, mouse monoclonal, F1804; Sigma), and 2 µl of anti-GFP tag antibody (2 mg ml^−1^, rabbit polyclonal, A-11122; Thermo Fisher). We used the Bio-Rad CFX96 system to analyze the input or IP DNA by quantitative PCR. See Supplementary Table 3 for the primer sequences.

### mRNA-Seq analysis

We extracted total RNAs and isolated mRNAs from wt and *ies6*Δ cells using poly(A)beads (NEXTflex). The stranded libraries were generated from biological replicates of each strain using NEXTflex Rapid Directional RNA-Seq Kit according to the manufacturer’s manual and sequenced as single end. The sequence reads were aligned using STAR aligner^53^. Cufflinks tools were used to assemble the transcripts, to calculate their abundance in FPKM, and to determine list of genes that are differentially expressed in *ies6*Δ cells compared to wt. Genes whose expressions were increased or decreased more than twofold in *ies6*Δ cells relative to wt with *q*-value (false discovery rate-adjusted *p*-value) of ˂0.05 were defined as significantly upregulated or downregulated genes, respectively (Supplementary Table 1).

### Generation of silencing-defective alleles of *ino80*^+^

To identify mutant alleles of *ino80* ^+^, we used a Gene Morph II random mutagenesis kit (Stratagene) to mutagenize a DNA fragment containing the terminal 1.4 kb of the *ino80* ^+^ open reading frame (ORF), which covered a significant portion of the catalytic domain. We then used fusion PCR to fuse the mutagenized fragments to a *kan* ^*R*^ marker gene and sequences downstream of the *ino80* ^+^ ORF. We selected transformants on G418-containing plates by screening for slow growth at 35.5 °C. Then, we further screened these mutants for defective silencing of *cnt1:ura4* ^+^ at different temperatures (25, 30, 32.5, 35.5 °C). This technique allowed us to isolate several cold-sensitive silencing-defective mutant alleles, including *ino80-11*. We identified the respective causative mutations in selected each strain using standard Sanger sequencing.

### Minichromosome loss assay

We used genetic crossing to introduce a non-essential minichromosome (chromosome 16 (Ch16)). Ch16 bears the *S. cerevisiae LEU2* gene, which allows it to complement *leu1-32*. We cultured wt and mutant cells containing Ch16 in minimal medium lacking leucine (PMG-Leu) to maintain the minichromosome. We plated these cells on non-selective (YES) plates with or without allowing them to divide 10 to 12 times in non-selective medium (YES). We then replica-plated the resulting colonies onto selective medium lacking leucine (PMG-Leu) and counted those that could and could not grow on selective medium (PMG-Leu). We calculated the rate of minichromosome loss using the following formula: loss rate per division (%)=100 × (1−(*F*/*I*)^1/*N*^), where *F* is the final percentage of minichromosome-containing cells, *I* is the initial percentage of minichromosome-containing cells, and *N* is the number of generations^54^. We tested three independent colonies for each strain. Data are presented as the mean loss rate±s.d. (error bars).

### Co-immunoprecipitation

Cell pellets from log-phase cultures were resuspended in NP40 lysis buffer (1% NP40, 200 mM NaCl, 2 mM EDTA, 6 mM Na_2_ HPO4, 4 mM NaH_2_PO_4_, 2 mM PMSF, protease inhibitors) and lysed by bead-beating. Extracts were immunoprecipitated with 25 µl anti-Flag (M2)-agarose (Sigma). Immunoprecipitates were subjected to western blotting for the presence of Ies6-5×Flag or CENP-A^Cnp1^ using horseradish peroxidase-conjugated anti-Flag (M2) antibody (1:5,000, A8592; Sigma) or affinity-purified anti-CENP-A^Cnp1^ antibody (1:1,000; in-house), respectively.

### Generation of tethering strains

We generated the *cnt1*:*bighyg* strain based on the *cnt1*:*ura4* ^+^ strain, which contains *ura4* ^+^ at the *Nco*I site of the central core region in centromere 1^23^. First, we used PCR to attach 2×*tetO* or 1×*tetO* to each end of a 1 kb DNA fragment derived from the internal coding region of *arg3* ^+^. We then integrated the resulting PCR fragments into the middle of *ura4* ^+^ and integrated the hygromycin-resistance gene (*hphMX6*), which is flanked by 2×*tetO*, into the center of the *arg3* ^+^ insertion. Finally, we deleted the endogenous *ura4* ^+^ and *arg3* ^+^ genes of the resulting *cnt1*:*bighyg* strain. The *ura4* ^+^ terminator sequence at the end of *cnt1:bighyg* was excluded from the analysis because it is also present at the end of the endogenous *ura4* ^+^ locus. We generated the TetR-2×Flag-Iec1 fusion strain by replacing the start codon (ATG) of the endogenous *iec1* ^+^ gene with a DNA fragment encoding TetR-2×Flag. We generated the TetR-2×Flag strain without the Iec1 fusion by fusing the *iec1* ^+^ promoter and the TetR-2×Flag sequence and integrating them into an ectopic *ura4* ^+^ locus to replace the promoter and coding region of *ura4* ^+^. For the Ino80-tethering experiments, we replaced the promoter and coding region of *ura4* ^+^ with the thiamine-repressible *nmt81* promoter and a DNA sequence encoding TetR-2×Flag-Ino80, TetR-2×Flag-Ino80^K873A^, or TetR-2×Flag. We induced expression of the fusion proteins by growing the strains in PMG medium lacking thiamine. We generated the TetR fusion gene-containing strains and *cnt1*:*bighyg* by genetic crosses and verified them using diagnostic PCR.

### Data availability

The raw and processed sequencing data from this publication have been submitted to the NCBI Gene Expression Omnibus (GEO; http://www.ncbi.nlm.nih.gov/geo/) under accession number GSE99589. The authors declare that all the data supporting the findings of this study are available within the article and its Supplementary Information files and from the corresponding author on reasonable request.

## Electronic supplementary material

Supplementary Information

Peer Review Files
