# Supplementary material for: The Ino80 complex mediates epigenetic centromere propagation via active removal of histone H3
Source: Nat Commun. 2017 Sep 13;8:529. doi: 10.1038/s41467-017-00704-3 (PMC5597579; doi:10.1038/s41467-017-00704-3)
Supplement: Supplementary file 1 — Supplementary Information [file 41467_2017_704_MOESM1_ESM.pdf]

Description of Supplementary Files

File Name: Supplementary Information
Description: Supplementary Figures and Supplementary Tables

File Name: Peer Review File

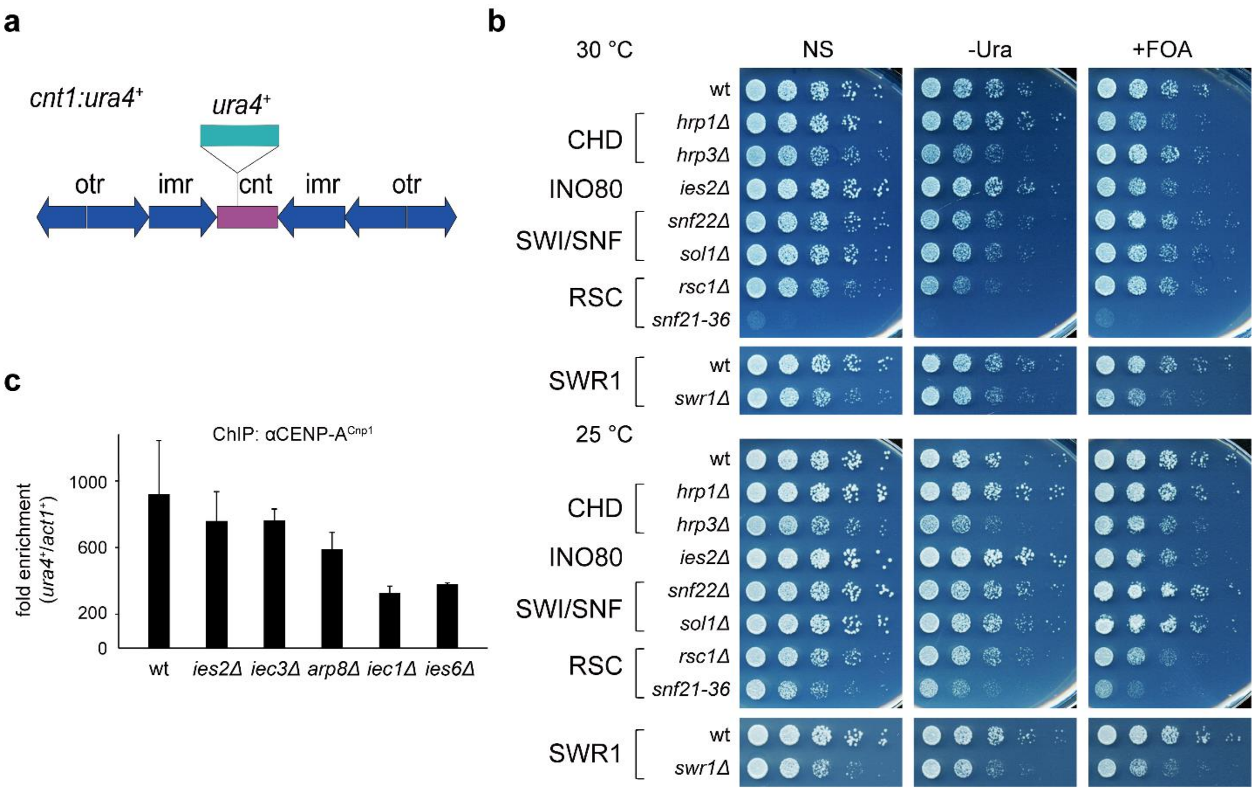

Supplementary Figure 1

Identification of chromatin-remodeling factors whose mutations impair silencing of *cnt1:ura4*$^+$. (**a**) Schematic of fission yeast centromere 1, indicating the central core (*cnt*), innermost repeat (*imr*), and outer repeats (*otr*). The *ura4*$^+$ insertion at centromere 1 (*cnt1*:*ura4*$^+$) is indicated by a box. (**b**) Silencing of *cnt1*:*ura4*$^+$ in wild-type (wt), *hrp1*Δ, *hrp3*Δ, *ies2*Δ, *snf22*Δ, *sol1*Δ, *rsc1*Δ, *snf21-36*, and *swr1*Δ cells. Chd1^{Hrp1} and Chd1^{Hrp3} are fission yeast paralogs of Chd1. Ies2 is a component of the Ino80 complex. Snf22 and Sol1 are components of the SWI/SNF complex. Rsc1 and Snf21 are components of the RSC complex. Swr1 is a component of the SWR1 complex. Serial dilutions (5-fold) of cells were spotted onto non-selective (NS), uracil-depleted (-Ura), or FOA-containing (+FOA) media at 30 °C or 25 °C. FOA was used to kill *ura4*$^+$-expressing cells. The *snf21-36* cells are temperature-sensitive, and can grow only at 25 °C. (**c**) ChIP analysis for enrichment of CENP-A^{Cnp1} at *ura4*$^+$ inserted within *cnt1* (*cnt1:ura4*$^+$) in wt cells and those harboring mutants of the Ino80 complex (*ies2*Δ, *iec3*Δ, *arp8*Δ, *iec1*Δ, and *ies6*Δ). Fold enrichment was calculated by comparing the *ura4*$^+$/*act1*$^+$ ratio between IP and input DNA. Data indicate the mean ± SD (error bars) for three biological replicates.

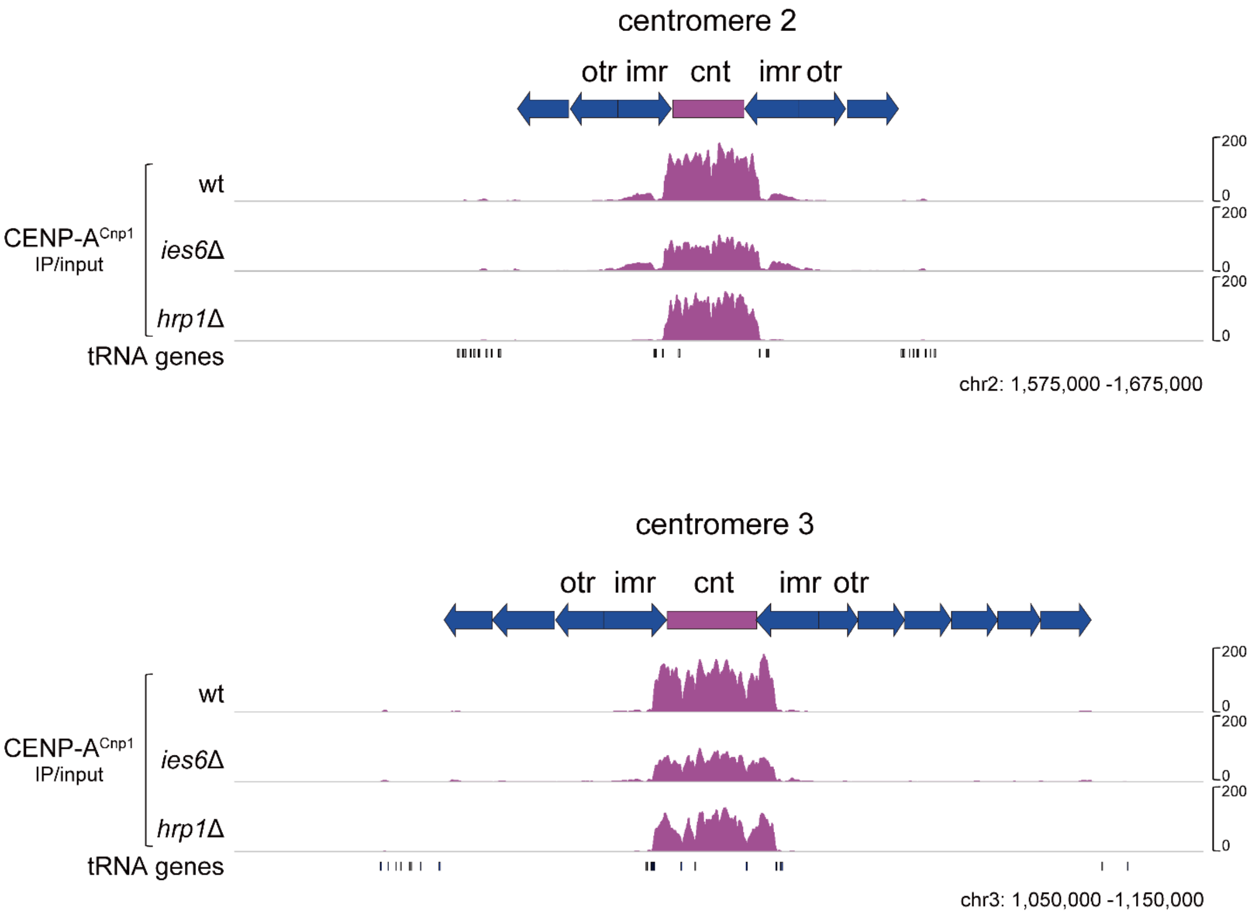

Supplementary Figure 2

Defective assembly of CENP-A^{Cnp1} chromatin at centromeres 2 and 3 in *ies6*Δ cells. Genome browser views of centromeres 2 and 3 showing the ChIP-Seq profiles of CENP-A^{Cnp1} (magenta) in wt, *iec6*Δ, and *hrp1*Δ cells. tRNA gene locations are indicated below (black tick marks).

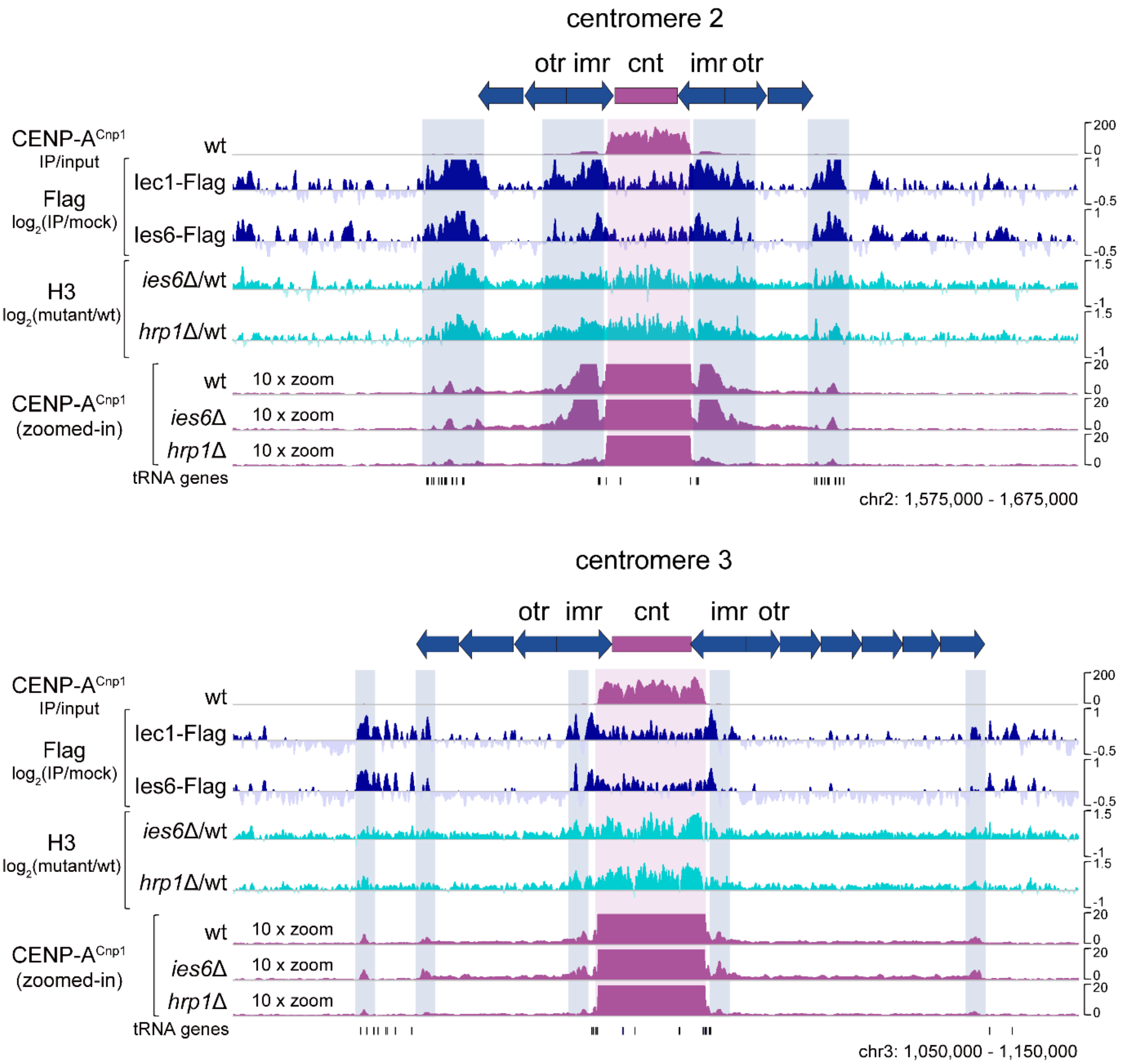

Supplementary Figure 3

Correlation between the localization of the Ino80 complex, its ability to remove H3-containing nucleosomes, and the assembly of CENP-A$^{\text{Cnp1}}$ nucleosomes at centromeres 2 and 3. Genome browser views of centromeres 2 and 3 showing the ChIP-Seq profiles of CENP-A$^{\text{Cnp1}}$, Iec1-5×FLAG, Ies6-5×FLAG, and the changes of H3 in *ies6*Δ or *hrp1*Δ cells relative to wt cells. The H3 ChIP-Seq experiments were performed and analyzed using wt *S. cerevisiae* cells as external spike-in controls (see Methods for more detail). Magnified (10×) views of CENP-A$^{\text{Cnp1}}$ in wt, *ies6*Δ, and *hrp1*Δ cells are shown below. The ChIP-Seq profiles of CENP-A$^{\text{Cnp1}}$ are the same as those in Supplementary Figure 2. The magenta vertical shading indicates the major CENP-A$^{\text{Cnp1}}$ peaks in the centromeres, while the blue vertical shading indicates the minor CENP-A$^{\text{Cnp1}}$ peaks in the heterochromatin (*imr*/*otr*) or at the heterochromatin/euchromatin boundaries. tRNA gene locations are indicated below (black tick marks).

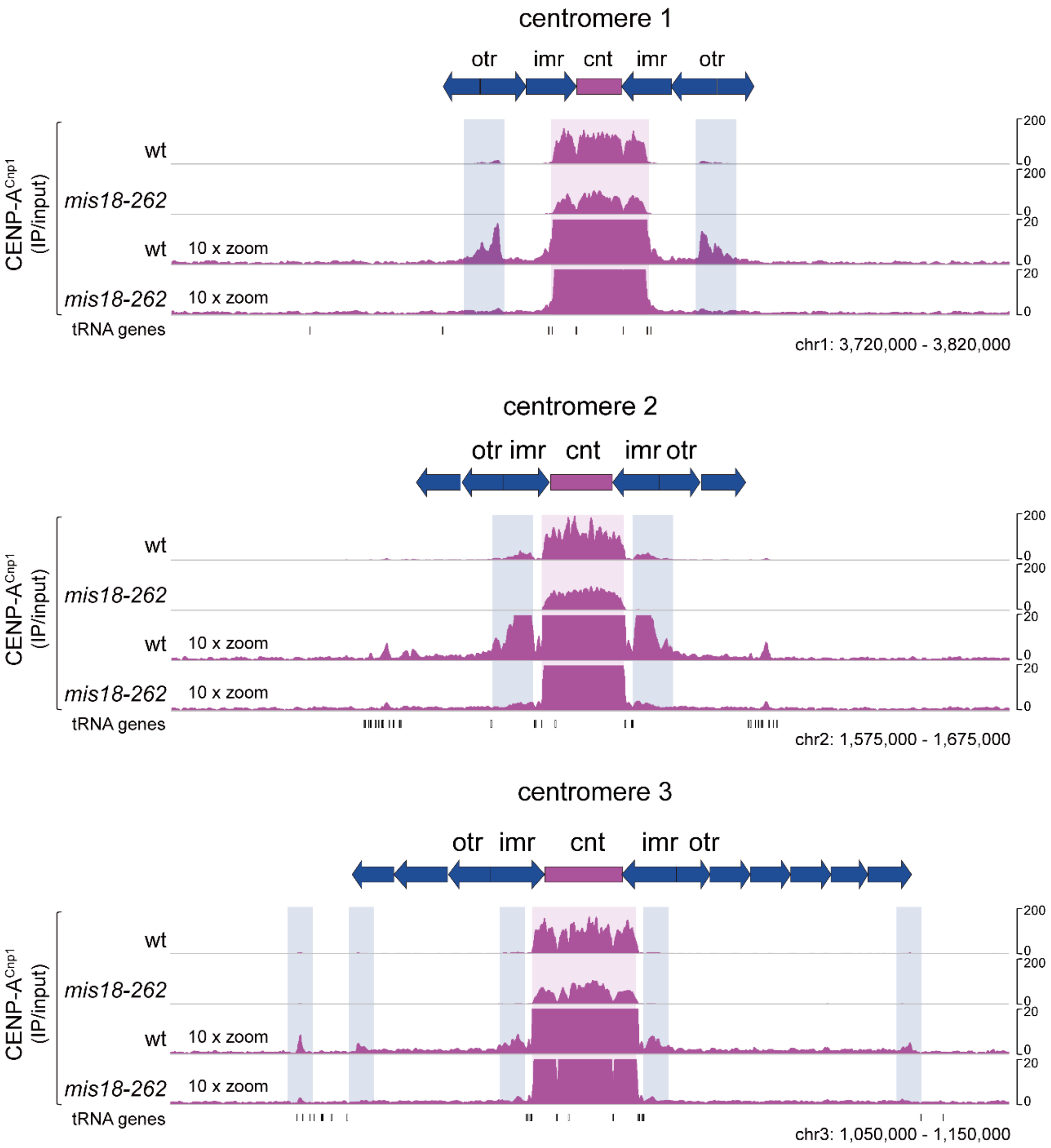

Supplementary Figure 4

Defective assembly of CENP-A^{Cnp1} nucleosomes at central domains and pericentromeric regions in *mis18-262* cells. Genome browser views of centromeres showing ChIP-Seq profiles of CENP-A^{Cnp1} in wt and *mis18-262* cells. Magnified (10×) views of CENP-A^{Cnp1} in wt and *mis18-262* cells are shown below. Cells were initially grown at 25 °C (permissive temperature for *mis18-262*) and then incubated at 36 °C (restrictive temperature for *mis18-262*) for 6 hours before harvest. The magenta vertical shading indicates the major CENP-A^{Cnp1} peaks in the centromeres, while the blue vertical shading indicates the minor CENP-A^{Cnp1} peaks in the heterochromatin (*imr*/*otr*) or at the heterochromatin/euchromatin boundaries. tRNA gene locations are indicated below (black tick marks).

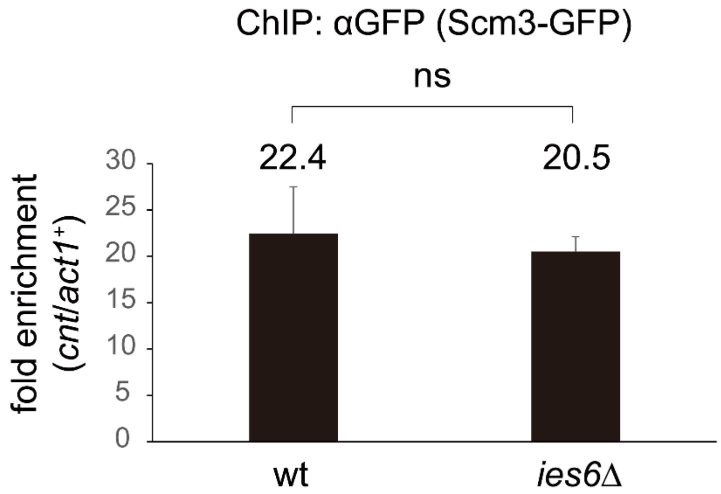

Supplementary Figure 5

The Ino80 complex does not affect the binding of Scm3 to the centromere. ChIP analysis for enrichment of Scm3-GFP at the central core region (*cnt*) in wt and *ies6*Δ cells. Fold enrichment was calculated by comparing the *cnt*/*act1*$^{+}$ ratio between IP and input DNA. Statistical significance was determined by Student's t-test; ns, not significant ($P > 0.05$).

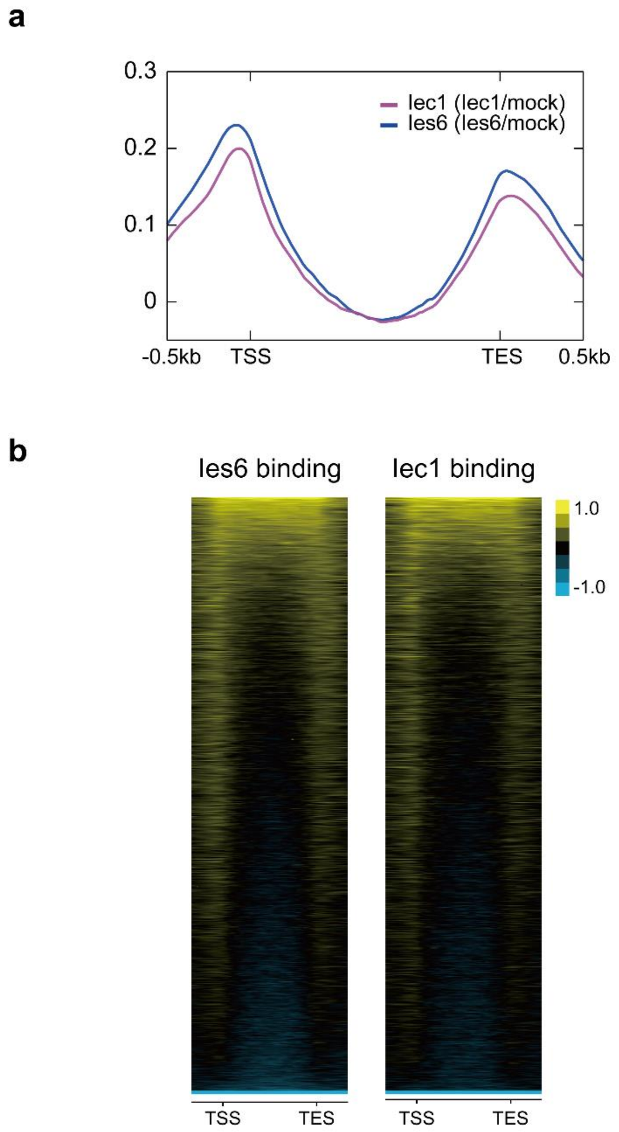

Supplementary Figure 6

Genome-wide correlation between Iec1 and Ies6 localization. (**a**) Average gene profiles of Iec1 binding (magenta) and Ies6 binding (blue). The y-axis indicates the $\log_2$ fold enrichment of Iec1-5×Flag and Ies5-5×Flag relative to that of the no tag control (mock). The profiles of Ies6 binding are the same as those in Figure 2c. TSS, transcription start site; TES, transcription termination site. (**b**) Heatmaps representing the bindings of Ies6 (left) and Iec1 (right) across all genes of *S. pombe* (from -0.5 kb of the TSS to +0.5kb of the TES; n = 7016). The heatmap for Ies6 binding is the same as that in Figure 2b. The heatmaps were sorted in descending order for Ies6 enrichment.

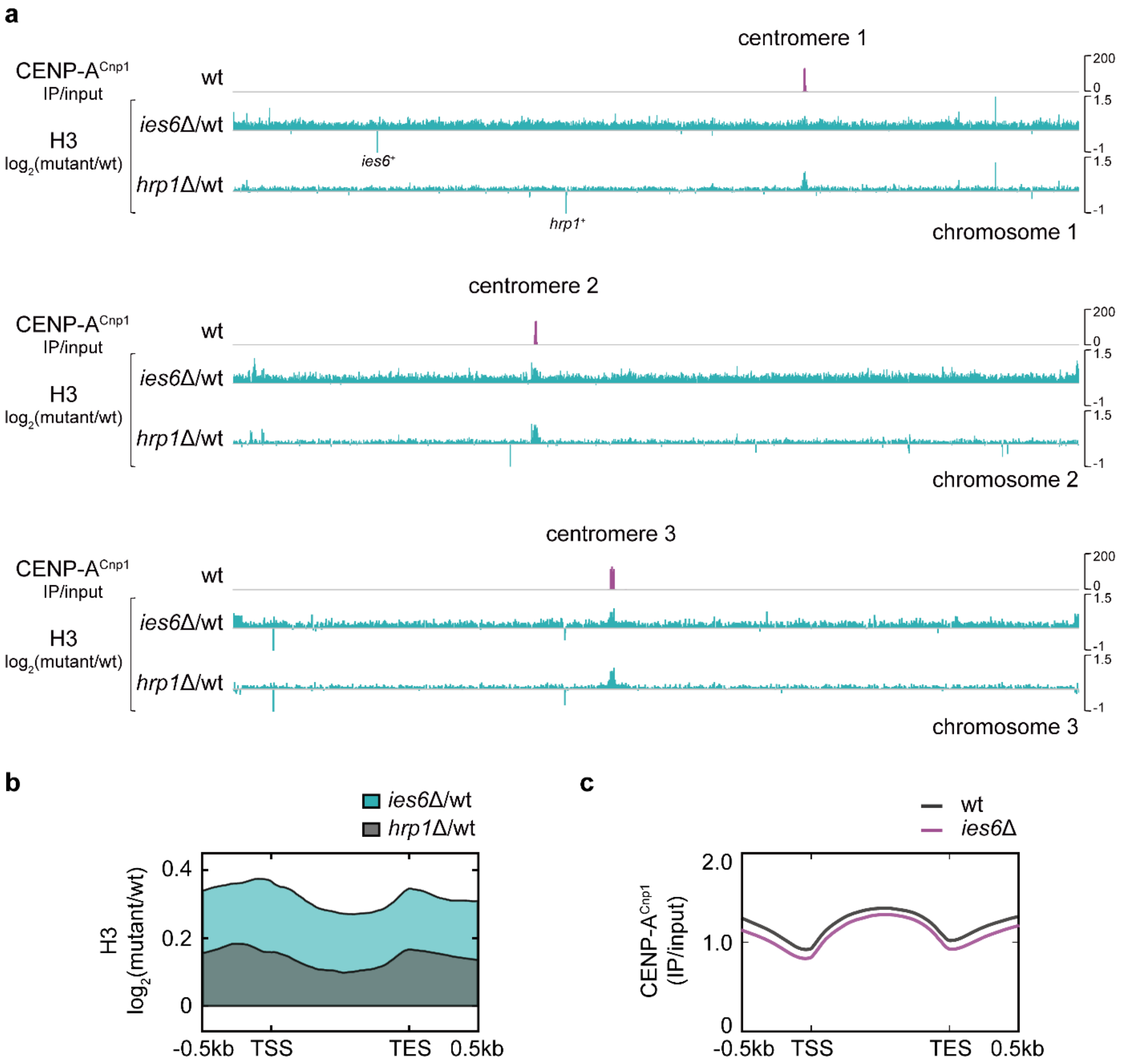

Supplementary Figure 7

The Ino80 complex globally acts at euchromatin to remove histone H3-containing nucleosomes but not CENP-A^{Cnp1} nucleosomes. (**a**) Genome browser views of chromosomes 1, 2, and 3 showing the ChIP-Seq profiles of CENP-A^{Cnp1} in wt cells, and H3 changes in *ies6*Δ and *hrp1*Δ cells. The H3 ChIP-Seq data are the same as those in Figure 2a. (**b**) Average gene profiles for H3 changes in *ies6*Δ and *hrp1*Δ cells. TSS, transcription start site; TES, transcription termination site. (**c**) Average gene profiles for CENP-A^{Cnp1} occupancies in wt (gray) and *ies6*Δ (magenta) cells. The CENP-A^{Cnp1} ChIP-Seq data are the same as those in Figure 2a.

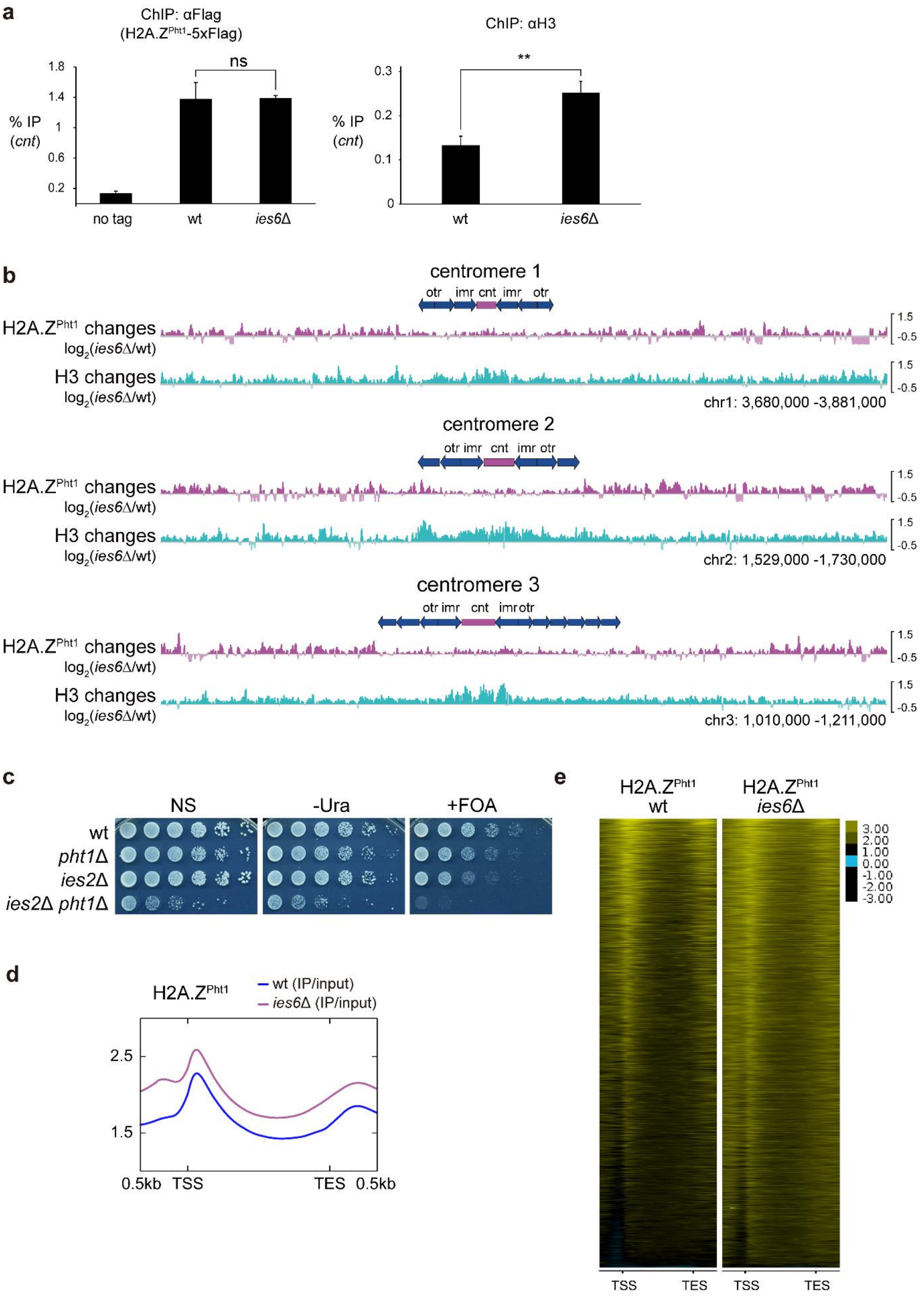
a
ChIP: αFlag
(H2A.Z^Pht1-5xFlag)
ns
% IP
(cnt)
no tag
wt
ies6Δ
ChIP: αH3
**
b
centromere 1
otr imr cnt imr otr
H2A.Z^Pht1 changes
log2(ies6Δ/wt)
H3 changes
log2(ies6Δ/wt)
chr1: 3,680,000 -3,881,000
centromere 2
chr2: 1,529,000 -1,730,000
centromere 3
chr3: 1,010,000 -1,211,000
c
NS
-Ura
+FOA
wt
pht1Δ
ies2Δ
ies2Δ pht1Δ
d
H2A.Z^Pht1
wt (IP/input)
ies6Δ (IP/input)
0.5kb TSS TES 0.5kb
e
H2A.Z^Pht1 wt
H2A.Z^Pht1 ies6Δ
TSS TES TSS TES

Supplementary Figure 8

The Ino80 complex regulates centromeric chromatin assembly independently of H2A.Z^{Pht1}. (**a**) ChIP analyses for the enrichments of H2A.Z^{Pht1}-5×Flag (left) and H3 (right) at the central core region (*cnt*) in wt and *ies6*Δ cells. The specificity of the H2A.Z^{Pht1}-5×Flag ChIP was confirmed by including wt cells with untagged H2A.Z^{Pht1} (no tag) as a negative control. ChIP enrichment (% IP) was calculated as the % ratio of IP relative to input. Statistical significance was determined by Student's t-test (ns, not significant; $P > 0.05$ and ** $P \leq 0.01$). (**b**) Genome browser views of 200-kb regions encompassing centromeres 1, 2, and 3, showing changes in H2A.Z^{Pht1} and H3 in *ies6*Δ relative to wt cells. The H3 ChIP-Seq data are the same as those in Figure 2a. Similar to the H3 ChIP-Seq experiments, the H2A.Z^{Pht1} ChIP-Seq experiments were performed and analyzed using wt *S. cerevisiae* cells expressing Sua7-5×Flag as an external spike-in control (see Methods for more detail). (**c**) Silencing of *cnt1*:*ura4*$^+$ in wt, *pht1*Δ, *ies2*Δ, and *ies2*Δ *pht1*Δ cells grown at 30 °C. Serial dilutions (5-fold) of cells were spotted onto non-selective (NS), uracil-depleted (-Ura), or FOA-containing (+FOA) medium. (**d**) Average gene profiles for the enrichments of H2A.Z^{Pht1} in wt (blue) and *ies6*Δ cells (magenta). TSS, transcription start site; TES, transcription termination site. Average gene profiles were generated using the protein-coding genes (n = 5156). (**e**) Heatmaps representing the enrichments of H2A.Z^{Pht1} in wt (left) and *ies6*Δ (right) cells across all protein-encoding genes of *S. pombe* (from -0.5 kb of the TSS to +0.5 kb of the TES; n = 5156). The heatmaps were sorted in descending order for enrichment of H2A.Z^{Pht1} in wt cells.

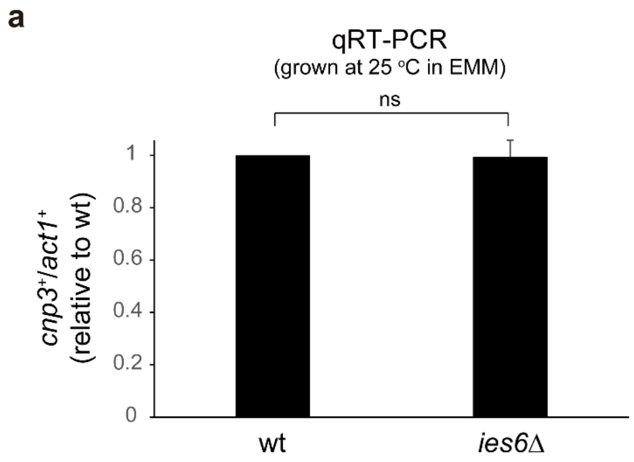

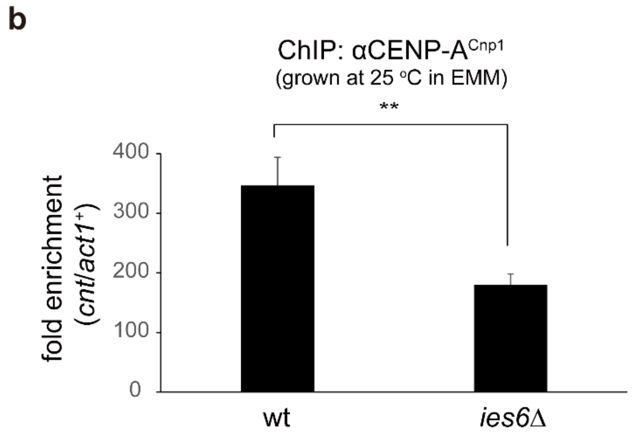

Supplementary Figure 9

The Ino80 complex regulates CENP-A^{Cnp1} chromatin assembly independently of its role in *cnp3*$^{+}$ expression. (**a**) qRT-PCR analysis of *cnp3*$^{+}$ expression in wt and *ies6*Δ cells grown under a suboptimal growth condition (at 25 °C in EMM). Expression of *act1*$^{+}$ served as a reference for normalization. Statistical significance was determined by Student's t-test; ns, not significant, $P > 0.05$. (**b**) ChIP analysis of CENP-A^{Cnp1} in the same cells. Statistical significance was determined by Student's t-test; ** $P \leq 0.01$.

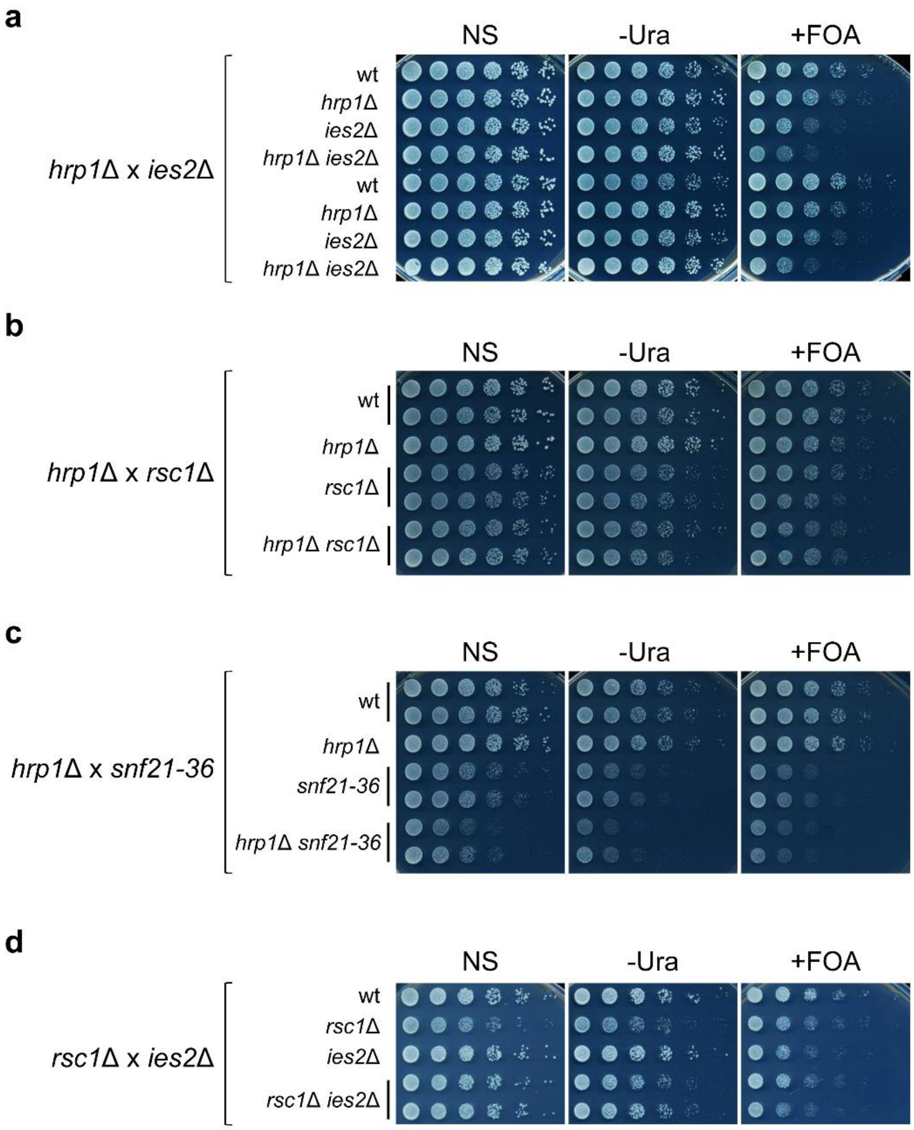

Supplementary Figure 10

The combination of *hrp1*Δ with *ies2*Δ but not *rsc1*Δ or *snf21-36* causes an additive defect in *cnt1*:*ura4*$^{+}$ silencing. (**a**) Silencing of *cnt1*:*ura4*$^{+}$ in wt, *hrp1*Δ, *ies2*Δ, and *hrp1*Δ *ies2*Δ cells. Serial dilutions (5-fold) of cells were spotted onto non-selective (NS), uracil-depleted (-Ura), or FOA-containing (+FOA) medium at 30 °C. FOA was used to kill *ura4*$^{+}$-expressing cells. (**b**) Silencing of *cnt1*:*ura4*$^{+}$ in wt, *hrp1*Δ, *rsc1*Δ, and *hrp1*Δ *rsc1*Δ cells grown at 30 °C. (**c**) Silencing of *cnt1*:*ura4*$^{+}$ in wt, *hrp1*Δ, *snf21-36*, and *hrp1*Δ *snf21-36* cells grown at the permissive temperature for *snf21-36* cells (25 °C). (**d**) Silencing of *cnt1*:*ura4*$^{+}$ in wt, *rsc1*Δ, *ies2*Δ, and *rsc1*Δ *ies2*Δ cells grown at 30 °C.

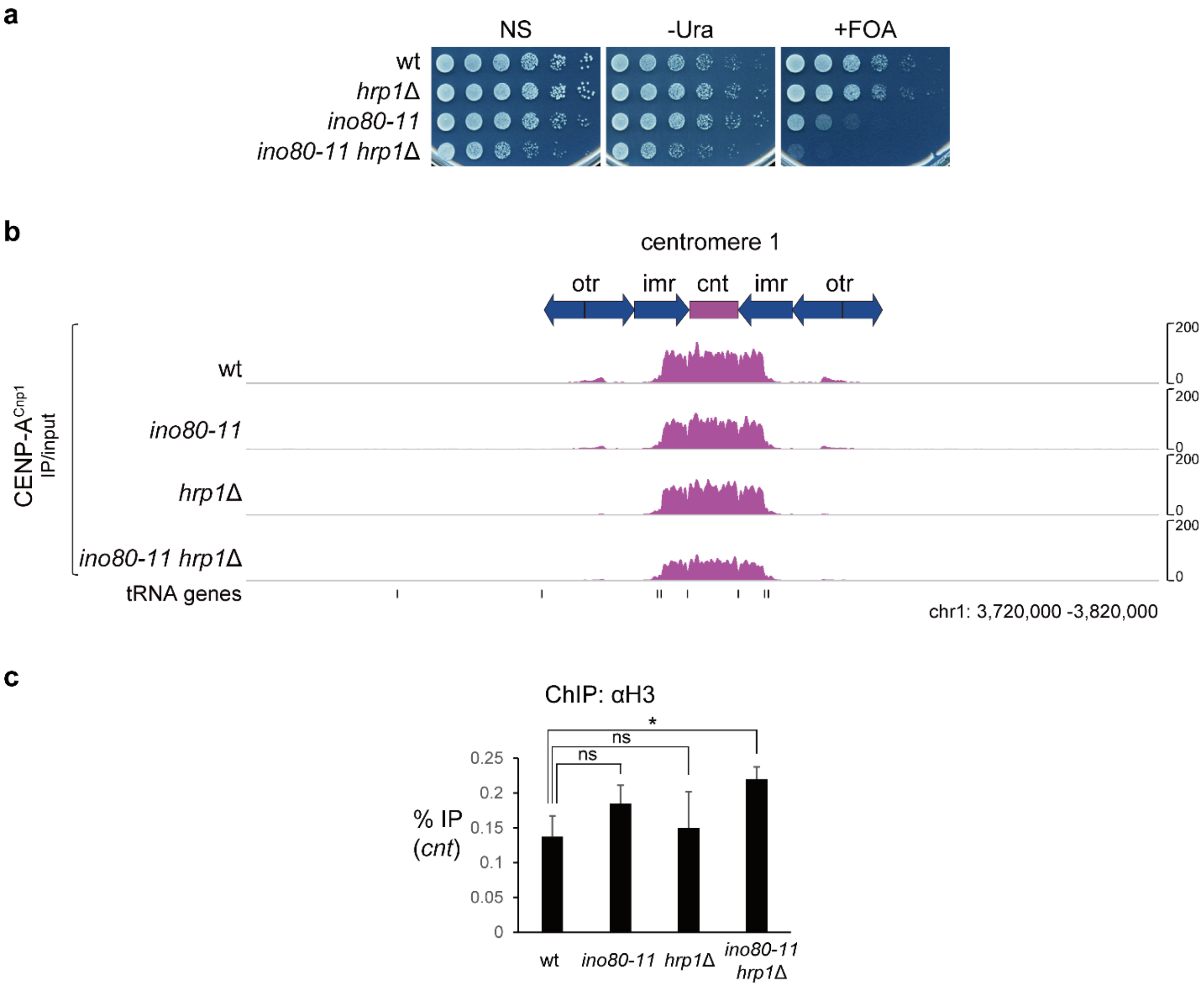

Supplementary Figure 11

The Ino80 complex and Chd1^{Hrp1} function redundantly in centromeric chromatin assembly. (**a**) Silencing of *cnt1*:*ura4*$^{+}$ in wt, *hrp1*Δ, *ino80-11***,** and *ino80-11 hrp1*Δ cells grown at 25 °C. Serial dilutions (5-fold) of cells were spotted onto non-selective (NS), uracil-depleted (-Ura), or FOA-containing (+FOA) medium. FOA was used to kill *ura4*$^{+}$-expressing cells. (**b**) Genome browser view of centromere 1 showing the ChIP-Seq profiles of CENP-A^{Cnp1} in wt, *ino80-11*, *hrp1*Δ, and *ino80-11 hrp1*Δ cells grown at the restrictive temperature (20 °C). (**c**) H3 ChIP analysis showing H3 occupancy at the central core region (*cnt*) in wt, *ino80-11*, *hrp1*Δ, and *ino80-11 hrp1*Δ cells. Data indicate the mean ± SD (error bars) for three biological replicates. The asterisk (*) denotes a statistically significant increase in H3 occupancy ($P \leq 0.05$), as determined by Student's t-test; ns, not significant ($P > 0.05$).

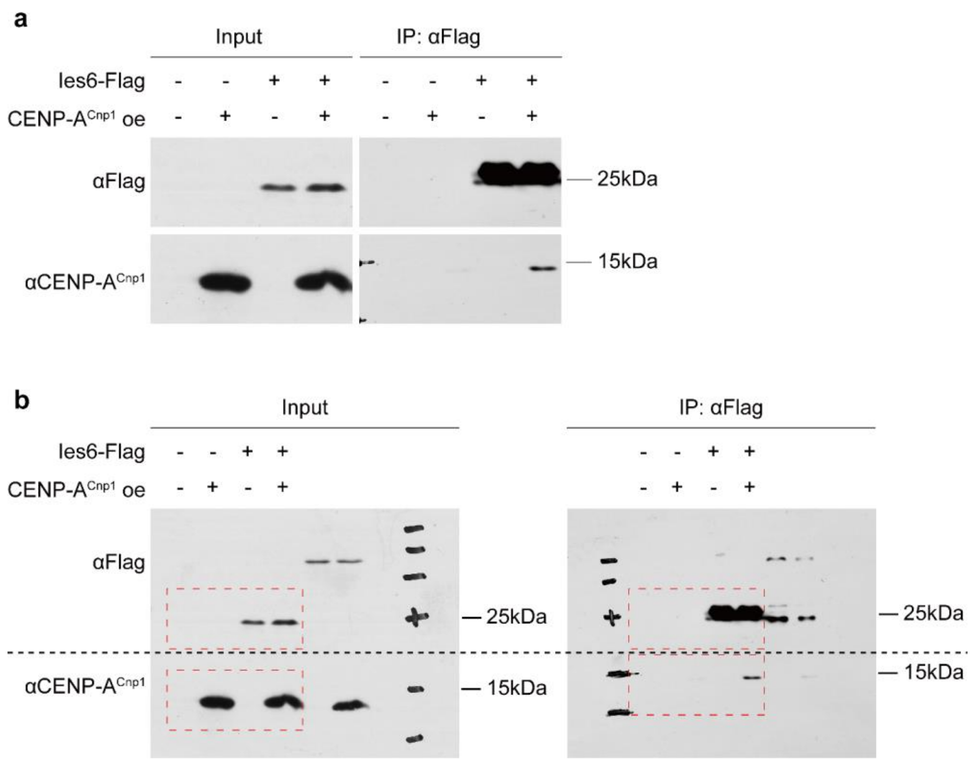

Supplementary Figure 12
Co-immunoprecipitation of Ies6-5×Flag and CENP-A$^{\mathbf{Cnp1}}$. (**a**) Whole-cell extracts obtained from strains with (+) or without (-) Flag-tagged Ies6 or overexpressed CENP-A^{Cnp1} (CENP-A^{Cnp1} oe; from the *nmt1* promoter) were immunoprecipitated with anti-Flag antibody. Input and immunoprecipitated (IP) samples were subject to Western blot analysis using the indicated antibodies. (**b**) Original and full blots for Supplementary Fig. 12a. The cropped regions were indicated by red-dotted line box. Membranes used for αFlag and αCENP-A^{Cnp1} Western blot analyses are separated by black-dotted line.

Genes whose mRNA levels are significantly down-regulated in *ies6*Δ cells (log2(Fold change) ≤ -1)

Systematic ID	Gene ID	log2(Fold change)	P value	Q value
SPAC1006.07	-	-1.01048	0.00005	0.00026645
SPAC1071.07c	rps1502	-1.1621	0.00005	0.00026645
SPAC11D3.02c	-	-1.66666	0.00005	0.00026645
SPAC11D3.04c	-	-1.13125	0.00005	0.00026645
SPAC11D3.05	mfs2	-1.51422	0.00005	0.00026645
SPAC11D3.06	-	-1.06651	0.0006	0.00246372
SPAC11G7.04	ubi1	-1.04324	0.00005	0.00026645
SPAC12G12.05c	taf9	-1.23259	0.00005	0.00026645
SPAC13D6.02c	byr3	-1.01114	0.00005	0.00026645
SPAC13G6.02c	rps101	-1.01017	0.00005	0.00026645
SPAC13G6.07c	rps601	-1.09947	0.00005	0.00026645
SPAC16A10.02	sub1	-1.0173	0.0021	0.00731061
SPAC1782.07	qcr8	-1.29051	0.00005	0.00026645
SPAC1782.11	met14	-1.1566	0.00005	0.00026645
SPAC1786.02	-	-1.1289	0.00005	0.00026645
SPAC17D4.01	pex7	-1.47993	0.00005	0.00026645
SPAC17G8.06c	-	-1.30446	0.00005	0.00026645
SPAC1834.01	sup45	-1.00852	0.00005	0.00026645
SPAC19G12.05	-	-1.42706	0.00005	0.00026645
SPAC1B2.06	-	-1.46094	0.00005	0.00026645
SPAC1B3.16c	vht1	-1.87751	0.00005	0.00026645
SPAC1F3.07c	rsc58	-1.08408	0.00005	0.00026645
SPAC1F7.07c	fip1	-1.73695	0.00005	0.00026645
SPAC1F7.08	fio1	-1.68927	0.00005	0.00026645
SPAC1F8.03c	str3	-2.96496	0.00005	0.00026645
SPAC222.04c	ies6	-1.26177	0.00005	0.00026645
SPAC222.18	-	-1.27788	0.00005	0.00026645
SPAC227.13c	isu1	-1.22091	0.0009	0.0035283
SPAC227.17c	-	-1.06575	0.0007	0.00282084
SPAC22E12.13c	rlp24	-1.04714	0.00005	0.00026645
SPAC23C4.14	alg1	-1.03539	0.00005	0.00026645
SPAC23H3.12c	-	-1.27238	0.00005	0.00026645
SPAC23H4.04	-	-1.17578	0.00005	0.00026645
SPAC23H4.09	cdb4	-1.09169	0.00005	0.00026645
SPAC24C9.13c	mrp10	-1.12879	0.00005	0.00026645
SPAC24H6.10c	-	-1.17052	0.00005	0.00026645
SPAC25B8.17	-	-1.26798	0.00005	0.00026645
SPAC25G10.05c	his1	-1.79914	0.00005	0.00026645
SPAC26A3.04	rpl2002	-1.35988	0.00005	0.00026645
SPAC26H5.10c	tif51	-1.07745	0.00005	0.00026645
SPAC27D7.07c	smd1	-1.37531	0.00005	0.00026645
SPAC27E2.03c	-	-1.11892	0.00005	0.00026645
SPAC27F1.06c	-	-1.2014	0.00005	0.00026645
SPAC2H10.02c	-	-1.03808	0.00005	0.00026645

SPAC30D11.09	cwf19	-1.34155	0.0002	0.00094419
SPAC343.12	rds1	-1.59237	0.00005	0.00026645
SPAC343.16	lys2	-1.23133	0.00005	0.00026645
SPAC3G6.05	-	-1.62458	0.00005	0.00026645
SPAC3G9.02	oar2	-1.13651	0.00005	0.00026645
SPAC3G9.03	rpl2301	-1.12225	0.00005	0.00026645
SPAC3G9.06	frs2	-1.09634	0.00005	0.00026645
SPAC3G9.10c	rrp41	-1.44529	0.00005	0.00026645
SPAC3H1.07	aru1	-1.23213	0.00005	0.00026645
SPAC3H5.05c	rps1401	-1.60535	0.00005	0.00026645
SPAC3H5.12c	rpl501	-1.05441	0.00005	0.00026645
SPAC458.02c	-	-1.26425	0.00005	0.00026645
SPAC4D7.13	usp104	-1.13613	0.00005	0.00026645
SPAC4G9.16c	rpl901	-1.25372	0.00005	0.00026645
SPAC513.03	mfm2	-1.19468	0.00005	0.00026645
SPAC521.05	rps802	-1.16387	0.00005	0.00026645
SPAC5H10.03	-	-2.07153	0.00005	0.00026645
SPAC644.05c	dut1	-1.3009	0.0001	0.00050046
SPAC664.04c	rps1602	-1.31249	0.00005	0.00026645
SPAC694.04c	-	-1.12302	0.00005	0.00026645
SPAC806.03c	rps2601	-1.39303	0.00005	0.00026645
SPAC821.08c	slp1	-1.31211	0.00005	0.00026645
SPAC821.09	eng1	-1.01973	0.00005	0.00026645
SPAC821.11	pro1	-1.00436	0.00005	0.00026645
SPAC8E11.10	-	-1.35075	0.00005	0.00026645
SPAC922.04	-	-1.27653	0.00005	0.00026645
SPAC922.06	-	-1.16433	0.0001	0.00050046
SPAC926.08c	-	-1.02251	0.00005	0.00026645
SPAC977.11	-	-1.09334	0.00005	0.00026645
SPAC977.12	-	-2.47062	0.00005	0.00026645
SPAC977.17	-	-1.00531	0.00005	0.00026645
SPAP7G5.04c	lys1	-1.16387	0.00005	0.00026645
SPAP7G5.05	rpl1002	-1.11784	0.00005	0.00026645
SPAP8A3.07c	-	-1.12679	0.00005	0.00026645
SPAPB1A10.14	pof15	-1.03473	0.00005	0.00026645
SPAPB8E5.03	mae1	-1.5329	0.00005	0.00026645
SPAPYUG7.05	-	-1.10881	0.0001	0.00050046
SPBC1105.02c	lys4	-1.24434	0.00005	0.00026645
SPBC1105.03c	mrpl16	-1.25124	0.00005	0.00026645
SPBC1105.04c	cbp1	-1.03079	0.00005	0.00026645
SPBC11C11.07	rpl1801	-1.07354	0.00005	0.00026645
SPBC11C11.10	-	-1.25243	0.0052	0.0158886
SPBC12C2.07c	-	-1.17083	0.00005	0.00026645
SPBC13E7.04	atp16	-1.37734	0.00005	0.00026645
SPBC146.08c	tif1102	-1.09673	0.00035	0.00153414
SPBC14C8.19	tam10	-1.24424	0.0009	0.0035283

SPBC14F5.13c	pho8	-1.16849	0.00005	0.00026645
SPBC15C4.03	-	-1.13179	0.0002	0.00094419
SPBC1683.09c	frp1	-2.67501	0.00005	0.00026645
SPBC1703.13c	-	-1.04431	0.00005	0.00026645
SPBC1711.04	-	-1.01715	0.00005	0.00026645
SPBC1711.05	-	-1.05518	0.00005	0.00026645
SPBC1734.01c	esf1	-1.01961	0.00005	0.00026645
SPBC17D1.04	acr1	-1.24199	0.0143	0.0374266
SPBC17D1.06	dbp3	-1.30652	0.00005	0.00026645
SPBC1861.01c	cnp3	-1.01117	0.00005	0.00026645
SPBC1861.02	abp2	-1.62668	0.00005	0.00026645
SPBC1921.07c	sgf29	-1.52709	0.00005	0.00026645
SPBC19C2.03	rpc10	-1.2109	0.00005	0.00026645
SPBC19C7.07c	sen34	-1.25243	0.00055	0.00228148
SPBC19F5.04	-	-1.09849	0.00005	0.00026645
SPBC1A4.02c	leu1	-1.08052	0.00005	0.00026645
SPBC21B10.04c	nrf1	-1.83017	0.00005	0.00026645
SPBC21C3.08c	car2	-2.10413	0.00005	0.00026645
SPBC21C3.16c	spt4	-1.00926	0.00015	0.00072608
SPBC21H7.04	dbp7	-1.13449	0.00005	0.00026645
SPBC24C6.04	-	-1.02798	0.00005	0.00026645
SPBC25H2.09	-	-1.30532	0.00005	0.00026645
SPBC26H8.06	grx4	-1.54952	0.00005	0.00026645
SPBC27.01c	-	-1.25942	0.00005	0.00026645
SPBC29A10.16c	-	-1.01065	0.00005	0.00026645
SPBC2F12.07c	rpl802	-1.04921	0.00005	0.00026645
SPBC2G2.05	rpl1603	-1.28393	0.00005	0.00026645
SPBC30D10.03c	-	-1.38577	0.00005	0.00026645
SPBC30D10.05c	-	-1.24571	0.00005	0.00026645
SPBC32F12.15	tfb5	-1.16201	0.00855	0.0243067
SPBC32H8.01c	-	-1.02688	0.0024	0.00818394
SPBC336.10c	tif512	-1.22734	0.00005	0.00026645
SPBC336.13c	-	-1.54378	0.0002	0.00094419
SPBC359.01	-	-1.8076	0.00005	0.00026645
SPBC359.02	alr2	-3.92525	0.00005	0.00026645
SPBC359.03c	aat1	-1.02595	0.00005	0.00026645
SPBC359.04c	pfl7	-1.84012	0.00005	0.00026645
SPBC36.03c	mfs3	-1.25163	0.00005	0.00026645
SPBC365.11	-	-1.01137	0.00925	0.0260347
SPBC3B8.03	-	-1.10038	0.00005	0.00026645
SPBC3B9.07c	rpa43	-1.26443	0.00005	0.00026645
SPBC3D6.08c	lsm1	-1.2375	0.00005	0.00026645
SPBC3E7.11c	-	-1.09059	0.00205	0.00716728
SPBC3H7.07c	ser2	-2.15904	0.00005	0.00026645
SPBC3H7.18	tam8	-1.27762	0.00005	0.00026645
SPBC409.08	-	-1.04816	0.00005	0.00026645

SPBC409.15	-	-1.06867	0.00005	0.00026645
SPBC418.01c	his4	-1.18578	0.00005	0.00026645
SPBC428.02c	eca39	-1.15694	0.00005	0.00026645
SPBC460.01c	-	-1.37977	0.00005	0.00026645
SPBC460.02c	-	-1.67422	0.00005	0.00026645
SPBC460.05	-	-1.81772	0.00005	0.00026645
SPBC4C3.07	-	-1.31223	0.00005	0.00026645
SPBC4F6.09	str1	-1.70976	0.00005	0.00026645
SPBC557.03c	pim1	-1.12929	0.0001	0.00050046
SPBC725.15	ura5	-1.52997	0.00005	0.00026645
SPBC83.15	-	-1.13646	0.00005	0.00026645
SPBC887.17	-	-1.32527	0.00005	0.00026645
SPBC8E4.01c	-	-2.1189	0.00005	0.00026645
SPBP22H7.08	rps1002	-1.13283	0.00005	0.00026645
SPBP23A10.03c	-	-1.01618	0.0002	0.00094419
SPBP23A10.15c	qcr1	-1.06328	0.00005	0.00026645
SPBP4G3.02	pho1	-1.48063	0.00005	0.00026645
SPBP4H10.15	-	-1.20218	0.00005	0.00026645
SPBP8B7.03c	rpl402	-1.00878	0.00005	0.00026645
SPBPB10D8.01	-	-1.89354	0.00005	0.00026645
SPBPB10D8.03	-	-1.36514	0.00195	0.00687687
SPBPB21E7.07	aes1	-2.08359	0.00005	0.00026645
SPBPB8B6.05c	-	-2.37689	0.00005	0.00026645
SPCC1183.08c	rpl101	-1.11113	0.00005	0.00026645
SPCC126.03	pus1	-1.27151	0.0017	0.0061283
SPCC126.11c	-	-1.60134	0.00005	0.00026645
SPCC1442.08c	cox12	-1.04022	0.0002	0.00094419
SPCC1682.14	rpl1902	-1.04974	0.00005	0.00026645
SPCC1739.05	set5	-1.08157	0.00005	0.00026645
SPCC1795.12c	-	-1.04265	0.00005	0.00026645
SPCC18.14c	rpp0	-1.09958	0.00005	0.00026645
SPCC1827.02c	-	-1.27847	0.00005	0.00026645
SPCC1827.06c	-	-1.00812	0.00005	0.00026645
SPCC1840.01c	mog1	-1.35663	0.00005	0.00026645
SPCC23B6.02c	-	-1.04957	0.00005	0.00026645
SPCC285.17	spp27	-2.11445	0.00005	0.00026645
SPCC330.03c	-	-1.19496	0.00005	0.00026645
SPCC330.07c	-	-1.58321	0.00005	0.00026645
SPCC330.10	pcm1	-1.21576	0.00005	0.00026645
SPCC330.14c	rpl2402	-1.06042	0.00005	0.00026645
SPCC4G3.06c	mrpl4	-1.86785	0.0004	0.00172537
SPCC4G3.17	-	-1.09041	0.00005	0.00026645
SPCC550.02c	cwf5	-1.1011	0.00005	0.00026645
SPCC550.11	-	-1.0282	0.00005	0.00026645
SPCC569.08c	ade5	-1.33926	0.00005	0.00026645
SPCC576.08c	rps2	-1.02544	0.00005	0.00026645

SPCC576.13	swc5	-1.87891	0.00065	0.00264396
SPCC794.12c	mae2	-1.3362	0.00005	0.00026645
SPCC962.04	rps1201	-1.03502	0.00005	0.00026645
SPCP1E11.09c	rpp103	-1.16333	0.00005	0.00026645
SPCP31B10.03c	med31	-1.12611	0.00005	0.00026645

Genes whose mRNA levels are significantly up-regulated in *ies6*Δ cells (log2(Fold change) ≥ 1)

Systematic ID	Gene ID	log2(Fold change)	P value	Q value
SPAC1002.01	-	1.06808	0.00025	0.00115096
SPAC1002.17c	urg2	3.83422	0.00005	0.00026645
SPAC1002.18	urg3	1.82141	0.00005	0.00026645
SPAC1002.19	urg1	6.17039	0.00005	0.00026645
SPAC1006.04c	mcp3	1.80504	0.00005	0.00026645
SPAC1039.01	-	1.04622	0.00005	0.00026645
SPAC1039.02	-	1.34629	0.00005	0.00026645
SPAC1039.09	isp5	2.88941	0.00065	0.00264396
SPAC1039.10	mmf2	1.68906	0.00445	0.013871
SPAC1093.07	-	1.03389	0.0015	0.00552381
SPAC10F6.15	-	4.80083	0.0092	0.0259164
SPAC11D3.01c	-	1.60156	0.00005	0.00026645
SPAC11G7.06c	mug132	1.49282	0.0005	0.00209548
SPAC11H11.01	sst6	1.18654	0.00005	0.00026645
SPAC11H11.04	mam2	1.66746	0.00005	0.00026645
SPAC1296.03c	sxa2	1.56682	0.0007	0.00282084
SPAC12G12.16c	-	1.50059	0.00005	0.00026645
SPAC139.05	-	3.09909	0.0076	0.0219811
SPAC1399.01c	-	1.71106	0.00005	0.00026645
SPAC1399.04c	-	1.67091	0.00005	0.00026645
SPAC13A11.03	mcp7	1.17499	0.00005	0.00026645
SPAC13A11.06	-	1.1638	0.00005	0.00026645
SPAC13D6.04c	btb3	1.10129	0.00735	0.0213531
SPAC13F5.01c	msh1	1.12092	0.00005	0.00026645
SPAC13G7.02c	ssa1	1.89154	0.00005	0.00026645
SPAC14C4.03	mek1	1.80769	0.00005	0.00026645
SPAC14C4.10c	-	1.39364	0.0029	0.00959674
SPAC1527.01	mok11	1.5686	0.00005	0.00026645
SPAC1565.07c	knd1	1.53512	0.003	0.00987234
SPAC15A10.05c	mug182	1.61639	0.00005	0.00026645
SPAC15A10.10	mde6	1.75755	0.00005	0.00026645
SPAC15E1.02c	-	3.30618	0.00005	0.00026645
SPAC15E1.10	-	1.15879	0.00005	0.00026645
SPAC15F9.01c	-	1.64627	0.00005	0.00026645
SPAC167.06c	mug143	1.49199	0.00005	0.00026645
SPAC167.08	Tf2-2	1.32267	0.00005	0.00026645
SPAC1687.23c	-	Inf	0.00005	0.00026645
SPAC16A10.08c	mug74	1.94423	0.00015	0.00072608
SPAC16E8.02	-	1.46558	0.0006	0.00246372
SPAC16E8.05c	mde1	2.80801	0.01695	0.0429603
SPAC1751.01c	gti1	2.20433	0.003	0.00987234
SPAC17A5.18c	rec25	1.65757	0.00005	0.00026645
SPAC17A5.19	-	1.49361	0.0003	0.00134493
SPAC17G8.13c	mst2	1.24684	0.00005	0.00026645

SPAC1805.03c	trm13	1.31458	0.00005	0.00026645
SPAC186.02c	-	1.66372	0.0088	0.0249193
SPAC186.04c	-	2.00378	0.00005	0.00026645
SPAC186.06	-	3.64433	0.00005	0.00026645
SPAC186.08c	-	4.57863	0.0019	0.0067408
SPAC186.09	-	5.62836	0.00435	0.0136247
SPAC18B11.03c	-	1.11474	0.0092	0.0259164
SPAC18B11.04	ncs1	1.64453	0.00005	0.00026645
SPAC1952.15c	rec24	1.88929	0.0009	0.0035283
SPAC19A8.16	prl65	1.39642	0.00005	0.00026645
SPAC19B12.10	sst2	1.10294	0.0009	0.0035283
SPAC19D5.07	uga1	2.19043	0.0026	0.00875562
SPAC19G12.04	dal1	1.36565	0.01425	0.0373108
SPAC19G12.09	-	1.96565	0.00005	0.00026645
SPAC19G12.10c	cpy1	1.10783	0.00005	0.00026645
SPAC1A6.01c	-	1.26082	0.00165	0.00597791
SPAC1A6.06c	meu31	1.15672	0.00035	0.00153414
SPAC1A6.08c	mug125	1.77686	0.01335	0.0353966
SPAC1B3.06c	-	1.36993	0.00005	0.00026645
SPAC1D4.03c	aut12	1.09432	0.00005	0.00026645
SPAC1F5.11c	tra2	1.01618	0.00205	0.00716728
SPAC1F7.10	-	1.55067	0.00005	0.00026645
SPAC1F7.12	yak3	1.53564	0.00005	0.00026645
SPAC1F8.01	ght3	1.71667	0.00005	0.00026645
SPAC1F8.05	isp3	1.03249	0.00005	0.00026645
SPAC20G4.02c	fus1	1.14823	0.0022	0.00760575
SPAC20G4.03c	hri1	1.78154	0.00005	0.00026645
SPAC20H4.04	fml2	1.29366	0.00005	0.00026645
SPAC212.06c	-	3.30525	0.0025	0.00848485
SPAC212.08c	-	2.90105	0.0028	0.00933238
SPAC222.15	meu13	3.45398	0.00005	0.00026645
SPAC22A12.17c	-	4.41782	0.0021	0.00731061
SPAC22F3.02	atf31	2.44038	0.00005	0.00026645
SPAC22F8.05	-	1.09178	0.00005	0.00026645
SPAC22G7.03	-	1.13543	0.01015	0.027962
SPAC22G7.07c	-	1.81905	0.00005	0.00026645
SPAC22G7.08	ppk8	1.16977	0.00295	0.00974235
SPAC22G7.11c	-	2.22813	0.00085	0.00336068
SPAC22H10.13	zym1	2.42499	0.00005	0.00026645
SPAC23D3.17	-	1.26606	0.00005	0.00026645
SPAC23G3.02c	sib1	1.65406	0.00005	0.00026645
SPAC23H3.15c	-	2.894	0.00005	0.00026645
SPAC23H4.05c	-	2.44519	0.00005	0.00026645
SPAC24C9.07c	bgs2	1.19429	0.00005	0.00026645
SPAC24C9.14	otu1	1.09911	0.00215	0.00746068
SPAC24C9.15c	spn5	3.50327	0.013	0.0345815

SPAC25B8.13c	isp7	2.00432	0.00005	0.00026645
SPAC25B8.20	-	1.08169	0.0048	0.0148339
SPAC25G10.02	cce1	1.62291	0.00005	0.00026645
SPAC25G10.04c	rec10	1.99281	0.00005	0.00026645
SPAC25H1.02	jmj1	1.29942	0.00005	0.00026645
SPAC26F1.04c	etr1	1.97488	0.00015	0.00072608
SPAC26F1.14c	aif1	1.58032	0.00005	0.00026645
SPAC26H5.08c	bgl2	1.41147	0.00005	0.00026645
SPAC26H5.09c	-	1.2904	0.00005	0.00026645
SPAC27D7.09c	-	2.89866	0.00005	0.00026645
SPAC27D7.11c	-	1.234	0.00005	0.00026645
SPAC27F1.05c	-	2.53587	0.00005	0.00026645
SPAC27F1.10	-	2.10637	0.0001	0.00050046
SPAC29A4.11	rga3	1.33954	0.00005	0.00026645
SPAC29A4.12c	mug108	4.35797	0.00075	0.003
SPAC29A4.13	-	1.18652	0.0012	0.00454266
SPAC29A4.19c	cta5	1.42521	0.00005	0.00026645
SPAC29B12.13	-	2.7184	0.00005	0.00026645
SPAC29E6.05c	mxr1	1.18805	0.00005	0.00026645
SPAC29E6.07	-	3.02498	0.00315	0.0103241
SPAC2E1P3.01	-	2.04009	0.00005	0.00026645
SPAC2E1P3.02c	amt3	2.35643	0.0185	0.0462571
SPAC2F3.05c	-	1.66518	0.00005	0.00026645
SPAC2G11.05c	rim20	1.48824	0.0001	0.00050046
SPAC30D11.02c	-	3.19613	0.0018	0.00643522
SPAC31G5.07	dni1	1.6073	0.0201	0.0496085
SPAC31G5.15	psd3	1.19563	0.01195	0.0321704
SPAC323.06c	uba5	1.12843	0.0144	0.0376428
SPAC32A11.02c	-	1.03749	0.00005	0.00026645
SPAC343.20	-	1.27464	0.00005	0.00026645
SPAC3A11.03	-	2.33513	0.00005	0.00026645
SPAC3A11.10c	-	1.00557	0.00005	0.00026645
SPAC3A12.02	-	1.08177	0.00005	0.00026645
SPAC3C7.05c	mug191	1.22297	0.00005	0.00026645
SPAC3C7.14c	obr1	1.62221	0.00005	0.00026645
SPAC3G6.07	-	3.09023	0.0066	0.0195502
SPAC3G9.11c	-	3.18597	0.00005	0.00026645
SPAC3H8.09c	nab3	1.31304	0.00005	0.00026645
SPAC4A8.04	isp6	1.18191	0.0001	0.00050046
SPAC4F10.08	mug126	2.5524	0.00005	0.00026645
SPAC4G9.05	mpf1	2.11029	0.0089	0.0251915
SPAC4G9.07	mug133	2.46833	0.00005	0.00026645
SPAC4G9.08c	rpc2	1.10884	0.00005	0.00026645
SPAC4H3.03c	-	3.04815	0.00005	0.00026645
SPAC4H3.04c	-	2.32772	0.00005	0.00026645
SPAC4H3.08	-	4.2607	0.00005	0.00026645

SPAC513.02	-	2.33989	0.00005	0.00026645
SPAC513.05	ams1	1.44654	0.00005	0.00026645
SPAC513.06c	-	2.71567	0.00005	0.00026645
SPAC513.07	-	1.47692	0.00005	0.00026645
SPAC56F8.13	-	1.50807	0.0057	0.017198
SPAC589.02c	med13	1.64047	0.00075	0.003
SPAC630.15	mug177	1.73973	0.00015	0.00072608
SPAC637.03	-	3.6743	0.00005	0.00026645
SPAC688.03c	-	1.58668	0.00005	0.00026645
SPAC688.06c	slx4	1.23888	0.0097	0.0269971
SPAC688.08	srb8	1.57203	0.00005	0.00026645
SPAC6B12.02c	mus7	1.0845	0.00005	0.00026645
SPAC6B12.06c	rrg9	1.32894	0.00025	0.00115096
SPAC6C3.07	mug68	4.48854	0.00005	0.00026645
SPAC6F12.14	cut23	1.1387	0.00115	0.00438147
SPAC6G10.03c	-	1.37731	0.00005	0.00026645
SPAC750.01	-	5.88348	0.00005	0.00026645
SPAC750.05c	-	3.53324	0.0005	0.00209548
SPAC869.01	-	2.99939	0.0054	0.0164148
SPAC869.02c	-	1.68392	0.00005	0.00026645
SPAC869.05c	-	1.51984	0.00005	0.00026645
SPAC869.06c	-	2.30867	0.0122	0.0327214
SPAC869.07c	mel1	4.76481	0.00025	0.00115096
SPAC869.08	pcm2	1.75678	0.00005	0.00026645
SPAC869.09	-	2.04004	0.00535	0.0163009
SPAC8F11.03	msh3	1.18711	0.00155	0.00567896
SPAC922.07c	atd2	1.34518	0.00005	0.00026645
SPAC922.09	-	3.67042	0.0173	0.0437109
SPAC959.05c	pdi4	1.52909	0.00335	0.010919
SPAC959.10	sen15	1.30039	0.0004	0.00172537
SPAC977.13c	-	2.49698	0.00005	0.00026645
SPAC977.18	-	3.31946	0.0011	0.00421321
SPAC9E9.01	-	1.48993	0.00005	0.00026645
SPAC9E9.09c	atd1	1.2639	0.00005	0.00026645
SPAC9E9.11	plr1	1.20817	0.00005	0.00026645
SPAC9G1.09	sid1	2.13857	0.0003	0.00134493
SPACUNK4.10	-	1.30827	0.0003	0.00134493
SPACUNK4.17	-	2.05047	0.00005	0.00026645
SPAP27G11.08c	meu32	2.22225	0.00005	0.00026645
SPAP7G5.03	prm1	2.05824	0.00245	0.00833257
SPAP7G5.06	per1	1.37664	0.00005	0.00026645
SPAP8A3.04c	hsp9	1.65018	0.00005	0.00026645
SPAPB17E12.09	-	2.71855	0.0056	0.0169356
SPAPB18E9.04c	-	2.01327	0.0077	0.0222308
SPAPB1A11.01	mfc1	2.34812	0.00005	0.00026645
SPAPB1A11.03	-	5.02959	0.0004	0.00172537

SPAPB24D3.07c	-	2.61344	0.00005	0.00026645
SPAPB24D3.08c	-	2.21444	0.00005	0.00026645
SPAPB24D3.10c	agl1	2.39215	0.00005	0.00026645
SPAPB2B4.04c	-	1.35552	0.00005	0.00026645
SPAPB8E5.08	-	2.87178	0.00045	0.0019156
SPAPJ691.02	-	2.92742	0.00005	0.00026645
SPBC119.03	-	2.43053	0.00005	0.00026645
SPBC119.04	mei3	3.48417	0.00005	0.00026645
SPBC119.05c	-	1.25653	0.00005	0.00026645
SPBC119.07	ppk19	1.08085	0.0004	0.00172537
SPBC119.16c	-	1.08542	0.00185	0.00658499
SPBC1198.01	fmd2	1.08907	0.00005	0.00026645
SPBC1198.04c	zas1	1.69261	0.00005	0.00026645
SPBC1198.14c	fbp1	3.10549	0.00005	0.00026645
SPBC11C11.04c	alp1	1.236	0.00005	0.00026645
SPBC1271.06c	mug96	Inf	0.00005	0.00026645
SPBC1271.07c	-	1.05156	0.00005	0.00026645
SPBC1271.08c	-	1.12653	0.00005	0.00026645
SPBC1271.09	-	1.09413	0.00005	0.00026645
SPBC1271.11	-	1.06399	0.00005	0.00026645
SPBC1289.14	-	4.33786	0.00005	0.00026645
SPBC1289.15	pfl5	1.37428	0.00005	0.00026645
SPBC1289.16c	cao2	1.77578	0.00005	0.00026645
SPBC1289.17	Tf2-11	1.85822	0.00005	0.00026645
SPBC1306.02	rtt10	1.1061	0.00005	0.00026645
SPBC1348.07	-	2.8889	0.0028	0.00933238
SPBC1348.12	-	1.3129	0.00005	0.00026645
SPBC1348.14c	ght7	1.48858	0.00255	0.0086275
SPBC13E7.09	vrp1	1.80332	0.00735	0.0213531
SPBC146.11c	mug97	3.41369	0.0003	0.00134493
SPBC15D4.02	gsf1	1.12894	0.00005	0.00026645
SPBC15D4.08c	-	1.52828	0.00955	0.0266596
SPBC15D4.12c	mug98	2.52984	0.00475	0.0146933
SPBC15D4.13c	-	1.08279	0.00035	0.00153414
SPBC1604.01	egt1	1.69545	0.00005	0.00026645
SPBC1683.02	-	1.37839	0.00515	0.0157581
SPBC1683.06c	-	2.07353	0.00005	0.00026645
SPBC1683.07	mal1	1.16486	0.00005	0.00026645
SPBC1685.06	cid11	3.88031	0.00005	0.00026645
SPBC1685.12c	-	1.5191	0.0026	0.00875562
SPBC1685.14c	-	1.50494	0.00005	0.00026645
SPBC16A3.02c	-	1.2998	0.00005	0.00026645
SPBC16A3.13	meu7	4.4715	0.00005	0.00026645
SPBC16D10.05	mok13	1.0163	0.00005	0.00026645
SPBC16E9.16c	lsd90	3.37184	0.00005	0.00026645
SPBC16G5.03	-	1.00151	0.00005	0.00026645

SPBC16H5.14c	-	1.31524	0.00005	0.00026645
SPBC1718.02	hop1	3.20284	0.0175	0.0440962
SPBC1773.03c	-	1.47794	0.00005	0.00026645
SPBC1773.05c	tms1	2.07622	0.0001	0.00050046
SPBC1773.06c	adh8	3.19881	0.00005	0.00026645
SPBC1773.08c	omh4	1.39804	0.00005	0.00026645
SPBC1773.12	-	1.19141	0.00865	0.0245587
SPBC1773.13	-	1.30965	0.00005	0.00026645
SPBC1778.04	spo6	1.93319	0.00005	0.00026645
SPBC17D1.07c	-	1.7479	0.00355	0.0114162
SPBC17D11.03c	-	1.71437	0.00005	0.00026645
SPBC18H10.07	-	1.72429	0.00005	0.00026645
SPBC18H10.09	-	1.85365	0.00015	0.00072608
SPBC19C7.04c	-	3.00576	0.00005	0.00026645
SPBC19F8.01c	spn7	1.69276	0.00005	0.00026645
SPBC19F8.06c	meu22	2.9733	0.0004	0.00172537
SPBC1D7.05	byr2	1.46631	0.00045	0.0019156
SPBC21.02	rtc5	1.601	0.00215	0.00746068
SPBC21.07c	ppk24	1.22372	0.00355	0.0114162
SPBC215.11c	-	3.42934	0.00005	0.00026645
SPBC216.02	mcp5	1.71557	0.00005	0.00026645
SPBC21C3.01c	vps13a	1.3371	0.00955	0.0266596
SPBC21C3.19	-	1.38181	0.00005	0.00026645
SPBC21D10.06c	map4	1.98525	0.00005	0.00026645
SPBC21D10.08c	-	2.01367	0.0004	0.00172537
SPBC23E6.03c	nta1	1.79091	0.00005	0.00026645
SPBC23G7.10c	-	3.11139	0.00005	0.00026645
SPBC23G7.11	mag2	2.77326	0.0001	0.00050046
SPBC23G7.13c	-	2.76356	0.01035	0.0284046
SPBC24C6.09c	-	3.80788	0.00005	0.00026645
SPBC25H2.03	-	1.19481	0.00005	0.00026645
SPBC28E12.06c	lvs1	1.1707	0.0009	0.0035283
SPBC29B5.02c	isp4	1.37514	0.00005	0.00026645
SPBC2A9.02	-	1.66483	0.00005	0.00026645
SPBC2D10.14c	myo51	1.05549	0.00005	0.00026645
SPBC2F12.12c	cay1	1.27738	0.00005	0.00026645
SPBC2G2.10c	mug110	1.30472	0.00005	0.00026645
SPBC32H8.11	mei4	1.40711	0.00125	0.00471545
SPBC336.05c	-	2.49206	0.00005	0.00026645
SPBC337.11	-	1.1284	0.00005	0.00026645
SPBC354.11c	-	2.42009	0.00045	0.0019156
SPBC354.12	gpd3	1.82378	0.00005	0.00026645
SPBC359.06	mug14	1.99015	0.00135	0.0050487
SPBC365.12c	ish1	1.38661	0.01705	0.0431632
SPBC365.20c	pnc1	1.0792	0.01405	0.0368912
SPBC3E7.02c	hsp16	2.47092	0.00005	0.00026645

SPBC3E7.04c	-	1.08596	0.00005	0.00026645
SPBC4.01	dni2	2.79889	0.00005	0.00026645
SPBC428.07	meu6	3.26043	0.00005	0.00026645
SPBC428.08c	clr4	1.13931	0.00005	0.00026645
SPBC4C3.08	mug136	3.12436	0.00005	0.00026645
SPBC530.02	-	1.11225	0.00005	0.00026645
SPBC530.16	ksh1	1.16267	0.0059	0.0177191
SPBC56F2.05c	-	1.03961	0.00005	0.00026645
SPBC56F2.06	mug147	3.46951	0.00005	0.00026645
SPBC56F2.15	tam13	1.17846	0.00005	0.00026645
SPBC582.10c	-	1.13392	0.00815	0.0233226
SPBC609.01	-	1.41549	0.00065	0.00264396
SPBC609.04	caf5	2.63663	0.00005	0.00026645
SPBC646.17c	dic1	1.32664	0.00005	0.00026645
SPBC651.10	nse5	1.12288	0.00005	0.00026645
SPBC660.05	-	1.57155	0.00005	0.00026645
SPBC660.06	-	1.00027	0.00005	0.00026645
SPBC685.03	-	1.16252	0.00005	0.00026645
SPBC6B1.04	mde4	1.134	0.00005	0.00026645
SPBC713.12	erg1	1.42654	0.00005	0.00026645
SPBC713.13	-	1.8733	0.00005	0.00026645
SPBC725.03	-	1.74493	0.00005	0.00026645
SPBC725.06c	ppk31	2.81689	0.00005	0.00026645
SPBC725.10	-	2.10326	0.00005	0.00026645
SPBC83.12	-	1.8678	0.00005	0.00026645
SPBC839.06	cta3	2.6442	0.00005	0.00026645
SPBC887.06c	snx3	1.05271	0.00005	0.00026645
SPBCPT2R1.04c	-	1.10058	0.00065	0.00264396
SPBP19A11.07c	-	1.11331	0.00005	0.00026645
SPBP22H7.05c	abo2	1.05746	0.00005	0.00026645
SPBP35G2.06c	nup131	1.11957	0.00005	0.00026645
SPBP4G3.03	-	5.58411	0.00005	0.00026645
SPBP4H10.10	-	2.3161	0.00005	0.00026645
SPBP8B7.27	mug30	1.18862	0.0005	0.00209548
SPBP8B7.29	-	1.19726	0.00005	0.00026645
SPBPB21E7.01c	eno102	2.95914	0.00005	0.00026645
SPBPB21E7.02c	-	Inf	0.00005	0.00026645
SPBPB21E7.04c	-	3.70291	0.00005	0.00026645
SPBPB21E7.11	-	2.48166	0.00005	0.00026645
SPBPB2B2.01	-	1.03161	0.00005	0.00026645
SPBPB2B2.02	mug180	1.12847	0.00005	0.00026645
SPBPB2B2.05	-	1.32495	0.00005	0.00026645
SPBPB2B2.08	-	2.01531	0.00015	0.00072608
SPBPB2B2.11	-	1.70719	0.00005	0.00026645
SPBPB2B2.12c	gal10	2.46215	0.00005	0.00026645
SPCC1183.09c	pmp31	1.04065	0.00005	0.00026645

SPCC1183.11	msy1	1.55243	0.00005	0.00026645
SPCC11E10.03	mug1	2.35703	0.00055	0.00228148
SPCC11E10.09c	-	2.12849	0.00005	0.00026645
SPCC1223.09	-	1.63896	0.00005	0.00026645
SPCC1223.12c	meu10	1.172	0.0004	0.00172537
SPCC1235.01	-	1.27937	0.00005	0.00026645
SPCC1235.08c	pdh1	1.01083	0.0016	0.00583582
SPCC1235.14	ght5	1.04257	0.00005	0.00026645
SPCC1235.17	-	1.37331	0.00005	0.00026645
SPCC1259.16	-	1.20039	0.00005	0.00026645
SPCC126.14	prp18	1.00407	0.0013	0.00488421
SPCC1281.07c	-	1.82604	0.00005	0.00026645
SPCC1281.08	wtf11	1.94976	0.00005	0.00026645
SPCC1322.06	kap113	1.28934	0.00005	0.00026645
SPCC1322.07c	mug150	1.04105	0.0092	0.0259164
SPCC1393.07c	mug4	1.95901	0.0039	0.0123643
SPCC1393.12	-	1.40857	0.00005	0.00026645
SPCC13B11.03c	-	2.36917	0.00005	0.00026645
SPCC13B11.04c	fmd3	1.96702	0.00005	0.00026645
SPCC1442.02	-	1.49339	0.00005	0.00026645
SPCC1442.07c	-	1.17934	0.00085	0.00336068
SPCC1450.09c	-	2.79164	0.00005	0.00026645
SPCC1450.13c	-	1.52145	0.00005	0.00026645
SPCC1620.03	mug163	1.79172	0.00005	0.00026645
SPCC162.03	-	1.66576	0.00005	0.00026645
SPCC162.10	ppk33	1.10811	0.0053	0.0161638
SPCC1682.08c	-	1.13777	0.00025	0.00115096
SPCC16A11.06c	gpi10	1.43186	0.00005	0.00026645
SPCC16A11.07	coq10	1.30626	0.00005	0.00026645
SPCC16A11.15c	-	1.30681	0.00005	0.00026645
SPCC1742.01	gsf2	1.70236	0.00005	0.00026645
SPCC1840.12	opt3	1.06399	0.02025	0.0499408
SPCC188.12	spn6	5.08983	0.00045	0.0019156
SPCC1884.01	-	1.6335	0.01505	0.0390434
SPCC1884.02	nic1	1.31393	0.00005	0.00026645
SPCC18B5.01c	bfr1	1.13388	0.00005	0.00026645
SPCC18B5.02c	-	5.6375	0.00005	0.00026645
SPCC18B5.05c	-	1.31934	0.00005	0.00026645
SPCC1906.04	wtf20	1.94306	0.00005	0.00026645
SPCC191.01	-	1.52511	0.00005	0.00026645
SPCC191.04c	-	2.81435	0.003	0.00987234
SPCC191.05c	-	1.92737	0.00005	0.00026645
SPCC191.06	-	1.93486	0.0008	0.00318042
SPCC191.09c	gst1	2.78592	0.00005	0.00026645
SPCC191.10	-	4.02346	0.0003	0.00134493
SPCC191.11	inv1	3.19587	0.00005	0.00026645

SPCC1919.05	-	1.18397	0.00005	0.00026645
SPCC24B10.14c	xlf1	1.43718	0.00005	0.00026645
SPCC285.05	-	1.90925	0.00005	0.00026645
SPCC2H8.02	-	1.15176	0.00005	0.00026645
SPCC306.10	wtf8	2.02496	0.00005	0.00026645
SPCC31H12.06	mug111	1.60525	0.00005	0.00026645
SPCC320.14	sry1	1.60383	0.00005	0.00026645
SPCC330.01c	rhp16	1.55442	0.00005	0.00026645
SPCC338.12	pbi2	2.38708	0.00005	0.00026645
SPCC338.18	-	1.79167	0.00005	0.00026645
SPCC417.02	dad5	1.32832	0.00265	0.00889174
SPCC417.11c	-	1.48364	0.00005	0.00026645
SPCC417.12	-	1.64589	0.015	0.0389448
SPCC4F11.04c	imt2	1.11462	0.0009	0.0035283
SPCC4G3.12c	-	1.07188	0.00005	0.00026645
SPCC548.03c	wtf4	1.06588	0.0095	0.0265885
SPCC550.07	-	1.1269	0.00005	0.00026645
SPCC569.02c	-	1.69394	0.00005	0.00026645
SPCC569.09	-	1.92727	0.00005	0.00026645
SPCC576.01c	-	1.87908	0.00005	0.00026645
SPCC576.02	-	1.58369	0.00005	0.00026645
SPCC576.16c	wtf22	1.16574	0.00365	0.0116915
SPCC584.03c	-	1.31533	0.00005	0.00026645
SPCC584.16c	-	1.10063	0.00005	0.00026645
SPCC5E4.04	cut1	1.0204	0.008	0.0229439
SPCC622.05	-	1.86574	0.00275	0.0091893
SPCC622.21	wtf12	1.18458	0.0024	0.00818394
SPCC63.04	mok14	1.03561	0.00005	0.00026645
SPCC663.03	pmd1	1.41365	0.00005	0.00026645
SPCC663.06c	osr1	5.36478	0.00005	0.00026645
SPCC663.07c	-	2.50694	0.0103	0.0283032
SPCC663.08c	-	5.75035	0.00005	0.00026645
SPCC70.12c	ecl1	1.33526	0.00005	0.00026645
SPCC736.13	-	1.05119	0.00005	0.00026645
SPCC74.09	mug24	2.55948	0.00005	0.00026645
SPCC757.02c	-	4.25765	0.0072	0.0210019
SPCC757.07c	ctt1	1.87098	0.00005	0.00026645
SPCC777.03c	-	2.37195	0.00005	0.00026645
SPCC777.04	-	5.46108	0.0002	0.00094419
SPCC794.01c	-	1.63242	0.0004	0.00172537
SPCC794.02	wtf5	1.70401	0.00005	0.00026645
SPCC794.04c	-	2.26361	0.00005	0.00026645
SPCC794.16	-	1.3867	0.0067	0.0197653
SPCC895.09c	ucp12	1.06532	0.00005	0.00026645
SPCC965.06	osr2	1.5035	0.00005	0.00026645
SPCC965.07c	gst2	4.20296	0.00005	0.00026645

SPCC965.08c	alr1	1.17107	0.00005	0.00026645
SPCC965.09	-	1.18561	0.00005	0.00026645
SPCC965.11c	agp3	1.72331	0.00005	0.00026645
SPCC965.14c	-	2.6239	0.00005	0.00026645
SPCC970.11c	wtf9	1.55631	0.00005	0.00026645
SPCP1E11.03	mug170	1.07301	0.01535	0.0397424
SPCP20C8.03	-	1.02821	0.00005	0.00026645
SPCPB16A4.06c	-	2.69353	0.00005	0.00026645
SPCPB1C11.01	amt1	1.53439	0.00005	0.00026645
SPCPB1C11.02	-	1.62274	0.00005	0.00026645
SPCPJ732.03	meu15	2.92952	0.0024	0.00818394

Changes in mRNA levels of CENP-A[Cnp1] chromatin-related genes in *ies6*Δ cells

Test ID	Gene ID	log2(Fold change)	P value	Q value	Significant (Q < 0.05)
SPBC1861.01c	cnp3	-1.01117	0.00005	0.000266448	yes
SPCC1672.10	mis16	-0.572672	0.0031	0.0101756	yes
SPBC36B7.08c	ccp1	-0.42183	0.0111	0.0301823	yes
SPBC18E5.03c	sim4	0.334732	0.01595	0.0410017	yes
SPAC1783.05	hrp1	0.471859	0.00505	0.0154959	yes
SPBC800.13	cnp20	0.476723	0.0044	0.0137481	yes
SPBC577.15c	sim3	0.484581	0.0005	0.00209548	yes
SPCC1393.04	fta4	0.67743	0.00005	0.000266448	yes
SPAC4F10.12	fta1	0.773097	0.00005	0.000266448	yes
SPBC776.16	eic2	0.788666	0.00005	0.000266448	yes
SPBP22H7.09c	mis15	0.885543	0.00005	0.000266448	yes
SPAC688.02c	mis14	-0.376556	0.03765	0.0831603	no
SPBC409.09c	mis13	-0.230173	0.21555	0.332513	no
SPBP8B7.12c	fta3	-0.140058	0.5105	0.6202	no
SPBC1105.11c	hht3	-0.119925	0.5912	0.692587	no
SPBC21.01	mis17	-0.101264	0.7855	0.851056	no
SPAC1834.04	hht1	-0.0274014	0.84325	0.89316	no
SPAPB1A10.02	scm3	-0.0153886	0.9626	0.975972	no
SPCC290.04	ams2	0.0356341	0.83165	0.884837	no
SPCC1235.07	fta7	0.0816815	0.79425	0.857478	no
SPBC409.04c	mis12	0.102005	0.6645	0.754253	no
SPCC970.12	mis18	0.128669	0.81405	0.872528	no
SPBC8D2.04	hht2	0.207339	0.2366	0.356099	no
SPBC1105.17	cnp1	0.23976	0.5525	0.657936	no
SPBC27B12.02	eic1	0.242868	0.2855	0.402825	no
SPAC1783.03	fta2	0.635883	0.115	0.207281	no
SPAC11H11.05c	fta6	1.01943	0.1521	0.257186	no
SPAC1687.20c	mis6	1.0846	0.15315	0.25854	no

Supplementary Table 1. Genes down- or up-regulated in *ies6*Δ cells and changes in mRNA levels of CENP-A[Cnp1] chromatin-related genes in *ies6*Δ cells

Strain	Genotype	Source
972	h^-	
1884	h^- cnt1:ura4$^+$ ade6-210 leu1-32 ura4-DS/E	R. Allshire
2586	h^- cnt1:ura4$^+$ hrp1Δ::nat ura4-DS/E	This study
2590	h^- cnt1:ura4$^+$ rsc1Δ::kan ura4-DS/E	This study
2593	h^- cnt1:ura4$^+$ sol1Δ::kan ura4-DS/E	This study
2597	h^- cnt1:ura4$^+$ snf21-36 ura4-DS/E	This study
2627	h^- cnt1:ura4$^+$ hrp3Δ::nat ura4-DS/E ade6-210 leu1-32	This study
2817	h^- cnt1:ura4$^+$ ies2Δ::kan ura4-DS/E	This study
4498	h^- cnt1:ura4$^+$ swr1Δ::kan ura4-DS/E	This study
2833	h^- cnt1:ura4$^+$ iec1Δ::nat ura4-DS/E	This study
2836	h^- cnt1:ura4$^+$ iec3Δ::kan ura4-DS/E	This study
2789	h^- cnt1:ura4$^+$ arp8Δ::nat ura4-DS/E	This study
2815	h^- cnt1:ura4$^+$ ies6Δ::nat ura4-DS/E	This study
1296	h^- ies6Δ:nat ade6-210 leu1-32 ura4-D18	This study
2941	h^- hrp1Δ:kan ura4-D18	Lab stock
1923	h^+ (Ch16 ade6-216 LEU2) ade6-210 leu1-32 ura4-D18*	R. Allshire
3810	h^+ (Ch16 ade6-216 LEU2) ies6Δ:nat ade6-210 leu1-32 ura4-D18	This study
4929	h^- (Ch16 ade-216 LEU2+) ies2Δ::kan ade6-210 leu1-32 ura4-D18	This study
4930	h^- (Ch16 ade-216 LEU2+) iec1Δ::nat ade6-210 leu1-32 ura4-D18	This study
4935	h^- (Ch16 ade-216 LEU2+) arp8Δ::nat ade6-210 leu1-32 ura4-D18	This study
4938	h^- (Ch16 ade-216 LEU2+) iec3Δ::nat ade6-210 leu1-32 ura4-D18	This study
2649	h^- cnt1:ura4$^+$ pht1Δ::nat ura4-DS/E	This study
2829	h^- cnt1:ura4$^+$ ies2Δ::kan pht1Δ::nat ura4-DS/E	This study
2217	h^- pht1-5×FLAG-hyg ade6-210 leu1-32 ura4-D18	This study
4709	h^- pht1-5×FLAG-hyg ies6Δ::nat ade6-210 leu1-32 ura4-D18	This study
2824	h^- cnt1:ura4$^+$ ies2Δ::kan hrp1Δ::nat ura4-DS/E	This study
2638	h^- cnt1:ura4$^+$ hrp1Δ::nat rsc1Δ::kan ura4-DS/E	This study
2642	h^- cnt1:ura4$^+$ hrp1Δ::nat snf21-36 ura4-DS/E	This study
4967	h^+ cnt1:ura4+ rsc1Δ::kan ies2Δ::nat ade6-210 leu1-32 ura4-D18orDS/E	This study
4965	h^- cnt1:ura4+ rsc1Δ::kan ies2Δ::nat ade6-210 leu1-32 ura4-D18 orDS/E	This study
3045	h^- cnt1:ura4$^+$ ino80-11-kan ade6-210 leu1-32 ura4-D18	This study
3066	h^- cnt1:ura4$^+$ ino80-11-kan hrp1Δ::nat ura4-DS/E	This study
3028	h^- Ies6-5×Flag-hyg	This study
3032	h^- Iec1-5×Flag-hyg	This study
1896	h^- mis18-262 ade6-210 his3-D1 arg3-D4*	R. Allshire
4923	h^- scm3-GFP-hyg	YGRC, Japan
4953	h^- scm3-GFP-hyg ies6Δ:nat	This study
3187	h^- cnt1:bighyg iec1P_TetR-2×Flag-iec1$^+$ ura4-D18 arg3-D4	This study
3194	h^- cnt1:bighyg ura4Δ::iec1P_TetR-2×Flag arg3-D4	This study
3200	h^+ cnt1:bighyg ura4Δ::nmt81P_TetR-2×Flag arg3-D4	This study
3683	h^+ cnt1:bighyg ura4Δ::nmt81P_TetR-2×Flag-ino80$^+$ arg3-D4	This study
3685	h^+ cnt1:bighyg ura4Δ::nmt81P_TetR-2×Flag-ino80-K873A arg3-D4	This study
4254	h^- leu1 ura4 Δcen1::Padh1-loxP-kanR cd60 (tel1R neocentromere)	K. Ishii
4281	h^- ies6-5×FLAG-hyg Δcen1::Padh1-loxP-kanR cd60 (tel1R neocentromere)	This study
1899	h- cnp1::ura4$^+$ lys1$^+$:cnp1-1 leu1-32 ura4$^-$*	R. Allshire
4188	h- ies6-5xFLAG-hyg cnp1Δ::ura4+ lys1:cnp1-1	This study
4192	h- iec1-5xFLAG-hyg cnp1Δ::ura4+ lys1:cnp1-1	This study

*Original source: M. Yanagida

Supplementary Table 2. List of strains used in our experiments.

Primer	Sequence	Target locus
qCnt1-fwd	CAGACAATCGCATGGTACTATC	*cnt*
qCnt1-rev	AGGTGAAGCGTAAGTGAGTG	*cnt*
qAct1-fwd	CCCAAATCCAACCGTGAGAAGATG	*act1*$^+$
qAct1-rev	CCAGAGTCCAAGACGATACCAGTG	*act1*$^+$
qCnp3-fwd	GGATTCGAGGATATGGATGC	*cnp3*$^+$
qCnp3-rev	ACTGCCATTGGAGACGTAGG	*cnp3*$^+$
qBighyg-1F	CAGCTAGAGCTGAGGGGATG	*bighyg*
qBighyg-1R	CGGATTTCTTCGTCAAATCG	*bighyg*
qBighyg-2F	CGCGGAGAGATTGTAAAGTTG	*bighyg*
qBighyg-2R	GCGACATTCTGAGACGACAA	*bighyg*
qBighyg-4F	GCGCCAGATCTGTTTAGCTT	*bighyg*
qBighyg-4R	AGTCACATCATGCCCCTGAG	*bighyg*
qBighyg-6F	GCAAACTGTGATGGACGACA	*bighyg*
qBighyg-6R	ACATTGTTGGAGCCGAAATC	*bighyg*
qBighyg-8F	TGGTCGCTATACTGCTGTCG	*bighyg*
qBighyg-8R	AGCAACCTTGGGGTCGTTA	*bighyg*
qBighyg-10F	AGCTCTTTGGCTACTGGTTCC	*bighyg*
qBighyg-10R	GGTTAGGAAATCGACGACCA	*bighyg*

Supplementary Table 3. List of primers used in our experiments
