# Supplementary material for: The Ino80 complex mediates epigenetic centromere propagation via active removal of histone H3
Source: Nat Commun. 2017 Sep 13;8:529. doi: 10.1038/s41467-017-00704-3 (PMC5597579; doi:10.1038/s41467-017-00704-3)
Supplement: Supplementary file 2 — Peer Review Files [file 41467_2017_704_MOESM2_ESM.pdf]

Reviewers' comments:

Reviewer #1 (Remarks to the Author):

Ino80C's cellular functions are not well understood so new information is important to the chromatin field. Ino80C has previously been implicated in H2AZ removal, gene activation, suppression of non-coding RNAs, DNA replication, repair and centromere function. Ino80C binds within heterochromatin and both up and downstream of genes in euchromatin in S. cerevisiae. Although this paper is a solid addition to the literature and shows to my satisfaction a significant effect of Ino80C in proper localization and accumulation of CENP-A at centromeres, binding of Ino80C to centromeres has been described in budding yeast albeit not to the same level of detail (i.e., older Seki lab papers, e.g., the 2007 Cell Cycle paper). Additionally, effects of Ino80C on centromere function, ploidy and fitness have been reported by Sue Biggins' and Jessica Downs' laboratories and certainly bear on the studies reported here but did not appear cited. There is a significant issue of scholarship that the authors must try harder to address.

Nevertheless, the data presented were by and large adequate to support the author's basic argument that CENP-A levels remain low and are ostensibly, with caveats, due to H3 remaining after replication. This should be straightforward to address biochemically and I was surprised the authors did not make such an attempt. Perhaps the issue of CENP-A chaperones, which was not well addressed, hindered a deeper analysis.

Interestingly, loss of Ino80 also leads to a mild increase in H3 up and downstream of the TSS and TTS, respectively. This could in principle be explained in part by Yen and Pugh's studies showing effects on nucleosome movement into the NDR in various Ino80C mutants. Indeed, the authors may be in a position to download Pugh's data in the mutants and see if their conclusions hold up in budding yeast. H2AZ removal tends to be a focus of additional uncited studies on Ino80C's effect at the centromere and the authors make a weak attempt to address this issue. They should take a look at Biggin's 2016 PLOS paper and tell us if they see CENP-A ectopic accumulation at promoters.

The technical strengths included the nice comparison of FOA sensitivity to molecular properties of the centromere, the neocen experiments, the solid ChIP analysis, the use of Ino80C catalytic mutants and the Tet-Ino80 tethering experiment described in Fig. 4. Much creativity here. Weaknesses included the failure to address numerous chromosome segregation issues that can lead to indirect effects and to accumulated issues in ploidy and fitness in deletions, the use of H3 ChIP that was not accompanied by MNase nucleosome positioning. Major issues in integrating previous literature.

Reviewer #2 (Remarks to the Author):

One of the most important areas of current investigation of eukaryotic chromosome biology is to uncover the molecular details that contribute to propagating the epigenetic mark that CENP-A nucleosomes generate to specify centromere location. The study by Choi et al. uses genetic and CHIP-sequencing data on cnt region in S.pombe regional centromeres to examine the role of chromatin remodelers on the loading and propagation of CENP-A in centromeric chromatin. The authors propose a model where H3 nucleosomes are exchanged with CENP-A by Ino80 complex. However, the actual role of Ino80 complex is not clearly demonstrated in this study, and many of the centromere experiments are similar in outcome to what has already been described in budding yeast by Chambers et al., 2012, G&D [a glaring omission from the work cited and discussed in this paper]. While Choi et al. document some small changes in chromatin composition and changes of CENP-A abundance in and around functional centromeres, there is not compelling enough evidence to support the primary claim of the paper for a direct role of a chromatin remodeling activity for

Ino80 in H3 histone exchange with CENP-A. Substantial existing structural and functional experiments from several labs strongly suggest that the Swr1/Ino80 complexes are primarily involved in the process of H2A.Z/H2B exchange and deposition, thus it remains more likely that the changes in H3 and CENP-A upon their manipulation are indirect, in my opinion. The authors' proposal would be intriguing, if correct, but with the limitations mentioned above, I do not think this study will be of strong interest to a broad readership within the centromere or chromatin remodeling fields. I list within my numbered comments, below, some suggestions for improving the existing data in the paper.

Suggestions to improve the existing experiments.
1. Most of the CHIP-sequence experiments were performed in the background of Ies6Δ. Deletion of this gene/Ino80 complex might affect diverse DNA organization processes, and as one observes in Fig.1A, the ies6Δ strain exhibits slow growth on normal media or -Ura dropout media compared to other Ino80 complex mutants. Addition of CHIP data from other Ino80 mutants that grow more normally would be helpful. The growth defect is unrelated to the centromere silencing defect, since the mutation of CENP-A itself (cnp1-87) has no effect on growth but a very strong silencing defect (Fig. 1A).
2. There is a growing understanding of the timing of CENP-A deposition during the fission yeast cell cycle, so experiments would be welcome to test at what stage(s) in the cell cycle the Ino80 complex is associated with cnt regions.
3. To relate to the known CENP-A chromatin assembly pathway, it will be important to test the extent to which Scm3 is localized to cnt region in Ino80 complex mutants.
4. The data in 3c are not compelling. Many studies have generated faulty conclusions based on CENP-A overexpression. Further, even if this interaction can be detected by an IP approach with endogenously expressed protein, it wouldn't support the conclusion by the authors that this interaction is meaningful. Unless additional controls/mutagenesis/etc are added.

Minor comments.
5. The paper would be improved for a general audience by additional introduction and discussion on what is already known about the specific functions of the Ino80 complex.
6. Related to Fig. S-1: SWR1 has a similar growth pattern to the hrp1Δ and ies2Δ. Does it have a similar decrease in CENP-A as seen in Fig. 1B/C with other mutants?
7. Related to Fig1: B: Please indicate the y-axis.
8. Related to Fig. S 4: does ies2/rsc12Δ have similar growth as ies2Δ, or does it further elevate 5-FOA sensitivity?

Reviewer #3 (Remarks to the Author):

Choi et al manuscript for Nature communications

This manuscript describes a role of the Ino80 chromatin remodeling complex at centromeres in fission yeast. The main findings are that mutations in genes encoding Ino80 subunits cause silencing defects at centromeres accompanied by reduced CENP-A levels, increased histone H3 levels and defects in chromosome segregation. Genome wide methodologies are used i.e. ChIP-seq to study global effects on H3 and CENP-A levels effects at a neo-centromere and using artificial tethering of Ino80 to non-centromeric DNA inserted into the centromere.

Points of concern
1. The possibility of indirect effects on centromere caused by mutations in Ino80 genes is not investigated. For example it is possible that Ino80 is required for expression of genes encoding CENP-A loading factors. This possibility should be ruled out by staying genome-wide gene expression changes in the mutants.
2. The silencing defects of iec1, ies2, iec3, arp8 and ies6 mutant strains is are not so clear from

the spotting assays (Fig 1a). There is only a slight reduction of growth on FOA plates. I suggest performing an RT-PCR analysis of the cnt1:ura4 reporter mRNA to strengthen this result.
3. TBZ sensitivity and chromosome loss measurements (Fig 1d) should be carried out for all iec1, ies2, iec3, arp8 and ies6.
4. The ChIP-seq analysis of H3 is not suitable for global quantification. It needs to be performed using DNA spiking controls adding the same amount to each sample to control for global changes, and also the results should be validated by Q-PCR at several loci. There are two problems with the analysis (described in the manuscript on pages 11-12). First the authors have used same amount of immunoprecipitated (IP) DNA from each sample for the library preparation and secondly the total number of reads have been used for normalization of each CHIP-seq sample. A global increase of H3 would lead to an increase of the IP material yielding more IP DNA and more sequence reads in total but this can not be detected by the current analysis. Therefore the conclusions made on page 5 about global effects of ies6 and hrp1 on H3 density are not valid.
5. The data shown in Supplementary Fig 3 are not conclusive since the double mutant strain is growing so slowly. A much better experiment is to test the H2AZ levels at centromeres in ies2 cells by ChIP.

Minor point
1. A reference to Buchanan et al 2009 should be added along with reference 17 on page 4 line 12.

Point-by-point response to reviewer's comments

Reviewer #1

Specific Comment:

Although this paper is a solid addition to the literature and shows to my satisfaction a significant effect of Ino80C in proper localization and accumulation of CENP-A at centromeres, binding of Ino80C to centromeres has been described in budding yeast albeit not to the same level of detail (i.e., older Seki lab papers, e.g., the 2007 Cell Cycle paper). Additionally, effects of Ino80C on centromere function, ploidy and fitness have been reported by Sue Biggins' and Jessica Downs' laboratories and certainly bear on the studies reported here but did not appear cited. There is a significant issue of scholarship that the authors must try harder to address.

Our Response:

In order to integrate and discuss the previous literature more properly, we have fully separated the **Introduction** and **Discussion** sections in the revised manuscript. According to the reviewer's suggestion, we cited the literature regarding the localization and function of the Ino80 complex at centromeres in *S. cerevisiae* (the Ogiwara et al., Chambers et al., and Hildebrand et al., papers) in the **Introduction** (p3, 4) and **Discussion** (p13, 14) sections. As noted by the reviewer, these *S. cerevisiae* studies reported roles of the Ino80 complex in regulating ploidy, centromere function, pericentric chromatin structure, and ectopic CENP-A^{Cse4} deposition at gene promoters. However, unlike our findings in *S. pombe*, the Ino80 complex in *S. cerevisiae* did not appear to regulate CENP-A chromatin assembly at the centromeres. We now discuss this discrepancy in the **Discussion** section (p13, 14) and hypothesize that the requirement for the Ino80 complex in the assembly of CENP-A nucleosomes at the centromeres might have been lost in budding yeast during the evolutionary transition from regional to point centromeres. Meanwhile, the connection of the Ino80 complex with centromeres and CENP-A was retained, allowing the emergence of divergent roles for the Ino80 complex in budding yeast.

Specific Comment:

Interestingly, loss of Ino80 also leads to a mild increase in H3 up and downstream of the TSS and TTS, respectively. This could in principle be explained in part by Yen and Pugh's studies showing effects on nucleosome movement into the NDR in various Ino80C mutants. Indeed, the authors may be in a position to download Pugh's data in the mutants and see if their conclusions hold up in budding yeast.

Our Response:

We carefully reviewed the findings reported by Yen and Pugh regarding the genome-wide localization of the Ino80 complex in *S. cerevisiae* (Yen et al., 2013). In this study, the authors showed that Ino80 subunits preferentially recognize nucleosome-free regions adjacent to +1 nucleosomes, which is similar to our current findings for the *S. pombe* Ino80 subunits, Ies6 and Iec1. We have included this point in the **Results** section (p7). Before comparing the roles of the Ino80 complex in nucleosome eviction observed in *S. pombe* with those reported for *S. cerevisiae*, we decided to more precisely analyze the changes of global histone H3 occupancy in the *S. pombe* Ino80 mutant. To achieve this, we performed quantitative histone H3 ChIP-Seq experiments in wild-type and *ies6*Δ cells, and included *S. cerevisiae* cells as external spike-in controls (described in the **ChIP-Seq and data analysis** subsection of the **Methods** section). In this new H3 ChIP-Seq analysis (**Fig. 2**, **Supplementary Fig. 3**, and **Supplementary Fig. 7**), the increases in histone H3 occupancy at the centromeres, gene TSSs, and gene TESs were even more significant than those shown in our previous H3 ChIP-Seq experiments, which were performed without the spike-in controls. Consistent with our finding that the *S. pombe* Ino80 complex acts to remove histone H3-containing nucleosomes at the TSS, Yen and Pugh also showed that the Ino80 complex in *S. cerevisiae* promotes full nucleosome turnover at +1 positions. We have included and discussed this point in the **Results** (p7) and the **Discussion** (p14) sections of the revised manuscript.

Specific Comment:

H2AZ removal tends to be a focus of additional uncited studies on Ino80C's effect at the centromere and the authors make a weak attempt to address this issue.

Our Response:

To address the reviewer's concern, we determined the role of the Ino80 complex in the removal of H2A.Z^{Pht1} from the centromere by performing ChIP analysis of H2A.Z^{Pht1} in wild-type and *ies6*Δ cells. In sharp contrast to the significant increase in H3 occupancy, *ies6*Δ did not significantly increase H2A.Z^{Pht1} occupancy at the central core region (**Supplementary Fig. 8a**). We also performed quantitative ChIP-Seq experiments for H2A.Z^{Pht1} with the inclusion of external spike-in controls. These experiments confirmed that the accumulation of H2A.Z^{Pht1} at the centromeres in *ies6*Δ cells is marginal when compared to the robust increase in H3 levels (**Supplementary Fig. 8b**). However, similar to the previous findings in *S. cerevisiae* (Papamichos-Chronakis, M. et al., 2011; Yen, K. et al., 2013), we observed that the Ino80 complex plays a prominent role in removing histone H2A.Z^{Pht1} from chromatin at gene promoters and coding regions (**Supplementary Fig. 8d, e**). These new analyses strengthen our conclusion that the Ino80 complex regulates centromeric chromatin assembly in a manner independent of its role in restricting H2A.Z^{Pht1} localization. We have included and discussed these results in the **Results** (p7, 8) and the **Discussion** (p14) sections of the revised manuscript.

Specific Comment:

They should take a look at Biggin's 2016 PLOS paper and tell us if they see CENP-A ectopic accumulation at promoters.

According to the reviewer's suggestion, we analyzed CENP-A^{Cnp1} accumulation at genic regions in *ies6*Δ cells (**Supplementary Fig. 7c**). However, unlike the findings reported for *S. cerevisiae*, we failed to observe CENP-A^{Cnp1} accumulation at gene promoters in *ies6*Δ cells. We speculate that this could reflect a functional divergence of Ino80 in *S. cerevisiae* that might have occurred during the evolution from regional to point centromeres. We now intensively discuss this point in the **Discussion** section (p13, 14).

Specific Comment:

Weaknesses included the failure to address numerous chromosome segregation issues that can lead to indirect effects and to accumulated issues in ploidy and fitness in deletions, the use of H3 ChIP that was not accompanied by MNase nucleosome positioning. Major issues in integrating previous literature.

Our Response:

1) To address the potential for indirect effects, we used mRNA-Seq analysis to compare the gene expression profiles of *ies6*Δ and wild-type cells. Consistent with the notion that the Ino80 complex plays a role in transcription, we identified hundreds of genes whose mRNA levels are significantly down- or up-regulated in *ies6*Δ cells (**Supplementary Table 1**). However, among the genes whose functions are known to be important for CENP-A^{Cnp1} chromatin assembly, only *cnp3*$^{+}$, which encodes the *S. pombe* ortholog of the CENP-A^{Cnp1}-binding protein, CENP-C^{Cnp3}, showed significant down-regulation in *ies6*Δ cells (**Supplementary Table 1**). Critically, we observed that *ies6*Δ does not cause a reduction of *cnp3*$^{+}$ expression under a suboptimal growth condition (at 25°C in minimal EMM) (**Supplementary Fig. 9a**), but *ies6*Δ cells still show significant reduction of CENP-A^{Cnp1} occupancy at the centromere under this condition (**Supplementary Fig. 9b**). Therefore, we conclude that the Ino80 complex regulates CENP-A^{Cnp1} chromatin regardless of its involvement in regulating *cnp3*$^{+}$ expression. We have included this in the **Results** section (p8, 9).

2) To exclude the possibility that the defective CENP-A^{Cnp1} chromatin assembly in *ies6*Δ cells is caused by complex secondary effects of *ies6*Δ that are unrelated to the function of Ino80, we performed ChIP analysis of CENP-A^{Cnp1} in other Ino80 mutants that grow more normally than *ies6*Δ cells (*iec1*Δ, *ies2*Δ, *iec3*Δ, and *arp8*Δ cells). From among these other mutations in the Ino80 complex, we found that *iec1*Δ clearly impairs CENP-A^{Cnp1} chromatin assembly, causes TBZ sensitivity, and dramatically increases minichromosome loss (**Supplementary Fig. 1c and Fig. 1b, d, e**). This analysis confirms that the function of the Ino80 complex is important for both centromere function and CENP-A^{Cnp1} chromatin assembly. We have included this in the **Results** section (p5).

3) Instead of adding a MNase-Seq analysis, which did not reliably yield quantitative results in our hands, we decided to improve our histone H3 ChIP-Seq data by performing quantitative

H3 ChIP-Seq experiments that included external spike-in controls (described in the **ChIP-Seq and data analysis** subsection of the **Methods** section). Compared to the previous data, the new data quantitative H3 ChIP-Seq data show more prominent increases in H3 levels at centromeres, gene TTSs, and gene TESs in *ies6*Δ cells (**Fig. 2, Supplementary Fig. 3, and Supplementary Fig. 7**). These improved results significantly strengthen our conclusion that the Ino80 complex plays a role in H3-nucleosome eviction at these genomic regions.

4) Finally, as suggested by the reviewer, we have integrated and discussed the previous literature (**References #18, #47, #48**).

Reviewer #2

Specific Comment:

However, the actual role of Ino80 complex is not clearly demonstrated in this study, and many of the centromere experiments are similar in outcome to what has already been described in budding yeast by Chambers et al., 2012, G&D [a glaring omission from the work cited and discussed in this paper].

Our Response:

According to the reviewer's suggestion, we cited the literature regarding the localization and function of the Ino80 complex at centromeres in *S. cerevisiae* (Chambers et al., Ogiwara et al., and Hildebrand et al.; **References #18, #47, #48**) in the **Introduction** (p3, 4) and **Discussion** (p13, 14) sections. As noted by the reviewer, these *S. cerevisiae* studies reported the roles of the Ino80 complex in regulating ploidy, centromere function, pericentric chromatin structure, and ectopic CENP-A^{Cse4} deposition at gene promoters. However, unlike our current findings in *S. pombe*, the Ino80 complex was not found to contribute to CENP-A chromatin assembly at the centromeres of *S. cerevisiae*. We now intensively discuss this discrepancy in the **Discussion** section (p13, 14) and offer our hypothesis that a requirement for the Ino80 complex in the assembly of CENP-A nucleosomes at centromeres might have been lost in budding yeast during the evolutionary transition from regional to point centromeres. However, the connection of the Ino80 complex with centromeres and CENP-A

was retained, allowing the emergence of divergent roles for the Ino80 complex in budding yeast.

Specific Comment:

While Choi et al. document some small changes in chromatin composition and changes of CENP-A abundance in and around functional centromeres, there is not compelling enough evidence to support the primary claim of the paper for a direct role of a chromatin remodeling activity for Ino80 in H3 histone exchange with CENP-A. Substantial existing structural and functional experiments from several labs strongly suggest that the Swr1/Ino80 complexes are primarily involved in the process of H2A.Z/H2B exchange and deposition, thus it remains more likely that the changes in H3 and CENP-A upon their manipulation are indirect, in my opinion.

Our Response:

1) To address the reviewer's concern that *ies6*Δ may cause only small changes in the chromatin composition at centromeres, we set out to improve our histone H3 ChIP-Seq data by performing quantitative H3 ChIP-Seq experiments that included external spike-in controls (described in the **ChIP-Seq and data analysis** subsection of the **Methods** section). Our new H3 ChIP-Seq data clearly demonstrate that *ies6*Δ prominently increases H3 levels across the central domains (**Fig. 2, Supplementary Fig. 3 and Supplementary Fig. 7**). This strengthens our conclusion that the Ino80 complex regulates the chromatin composition at centromeres by acting to remove H3-containing nucleosomes (p6).

2) To address the reviewer's concern that the Ino80 complex might regulate CENP-A^{Cnp1} chromatin by removing H2A.Z^{Pht1} from the centromeres, we performed ChIP analysis of H2A.Z^{Pht1} in wild-type and *ies6*Δ cells. We found that, in sharp contrast to the significant increase in H3 occupancy, *ies6*Δ does not cause a significant increase in H2A.Z^{Pht1} occupancy at the central core region (**Supplementary Fig. 8a**). We also performed quantitative ChIP-Seq experiments for H2A.Z^{Pht1} with the inclusion of external spike-in controls. Our findings confirmed that the accumulation of H2A.Z^{Pht1} at the centromeres in *ies6*Δ cells is marginal when compared to the robust increase in H3 levels (**Supplementary Fig. 8b**). However,

similar to the previous findings in *S. cerevisiae* (Papamichos-Chronakis, M. et al., 2011; Yen, K. et al., 2013), we observed that the Ino80 complex plays a prominent role in removing histone H2A.Z^{Pht1} from genic regions (**Supplementary Fig. 8b, d, e**). These new analyses strengthen our conclusion that the Ino80 complex regulates centromeric chromatin assembly in a manner independent of its role in restricting H2A.Z^{Pht1} localization. We have included and discussed these results in the **Results** (p7, 8) and **Discussion** (p14) sections.

Specific Comment:

Most of the CHIP-sequence experiments were performed in the background of Ies6Δ. Deletion of this gene/Ino80 complex might affect diverse DNA organization processes, and as one observes in Fig.1A, the ies6Δ strain exhibits slow growth on normal media or -Ura dropout media compared to other Ino80 complex mutants. Addition of CHIP data from other Ino80 mutants that grow more normally would be helpful. The growth defect is unrelated to the centromere silencing defect, since the mutation of CENP-A itself (cnp1-87) has no effect on growth but a very strong silencing defect (Fig. 1A).

Our Response:

As suggested by the reviewer, we performed ChIP analysis of CENP-A^{Cnp1} in Ino80 mutants that grow more normally than *ies6*Δ cells (*iec1*Δ, *ies2*Δ, *iec3*Δ, and *arp8*Δ cells) to exclude the possibility that the defective CENP-A^{Cnp1} chromatin assembly in *ies6*Δ cells is caused by complex secondary effects of *ies6*Δ that are unrelated to the function of Ino80. From among the other Ino80 complex mutants, *iec1*Δ clearly impaired CENP-A^{Cnp1} chromatin assembly, caused TBZ sensitivity, and dramatically increased minichromosome loss (**Supplementary Fig. 1c and Fig. 1b, d, e**). This analysis confirms that the function of the Ino80 complex is important for both centromere function and CENP-A^{Cnp1} chromatin assembly. We have included these findings in the **Results** section (p5).

Specific Comment:

There is a growing understanding of the timing of CENP-A deposition during the fission yeast cell cycle, so experiments would be welcome to test at what stage(s) in the cell cycle the Ino80 complex is associated with cnt regions.

Our Response:

As noted by the reviewer, it could be highly interesting to determine whether the association of the Ino80 complex with the central domains coincides with the timing of CENP-A^{Cnp1} deposition during the cell cycle. We attempted to address this question, but repeatedly encountered technical problems in performing ChIP analysis of the Ino80 complex in combination with cell cycle analysis. Because we needed to use a great number of cells (at least 2.4×10^9 cells per ChIP) to capture the weak chromatin binding of the Ino80 complex, the *cdc25-22* block-and-release experiment that we used for cell cycle analysis required us to perform cell cycle blockade and synchronous release of a massive amount of cells (~2 liters of starting volume was needed to enable cells to be collected at 8 time points). Unfortunately, we were unable to achieve a good cell cycle synchronization with this large-scale cell culture. In addition, the weak signals of the Ino80 subunit ChIP meant that we would need to normalize against mock (no tag) ChIP if we want to accurately quantify ChIP enrichments. This would require us to perform independent cell cycle analyses for the Ino80 subunit (Iec1 and Ies6) ChIP and mock (no tag) ChIP, which would add further inaccuracy to any determination of ChIP enrichment at a given cell cycle point. Given the above challenges, we would like to leave this question for a future study.

Specific Comment:

To relate to the known CENP-A chromatin assembly pathway, it will be important to test the extent to which Scm3 is localized to cnt region in Ino80 complex mutants.

Our Response:

According to the reviewer's suggestion, we performed ChIP analysis of Scm3-GFP in wild-type and *ies6Δ* cells. We found that the binding of Scm3 to the central core region (*cnt*) is not significantly impaired in *ies6Δ* cells (**Supplementary Fig. 5**). Since the association of

Scm3 with the centromeres requires kinetochore proteins (e.g., Sim4, Mis6, Mis16, and Mis18), but not CENP-A^{Cnp1} itself, this result indicates that the CENP-A^{Cnp1} deposition pathway is likely to be intact in *ies6Δ* cells. We have included this point in the **Results** section (p6, 7).

Specific Comment:

The data in 3c are not compelling. Many studies have generated faulty conclusions based on CENP-A overexpression. Further, even if this interaction can be detected by an IP approach with endogenously expressed protein, it wouldn't support the conclusion by the authors that this interaction is meaningful. Unless additional controls/mutagenesis/etc are added.

Our Response:

We agree with the reviewer that the physical interaction between CENP-A^{Cnp1} and Ies6 does not indicate any functional consequence of this interaction. Based on the widespread association of the Ino80 complex with CENP-A^{Cnp1} chromatin domains (**Fig. 2a**), we hypothesized that this physical interaction may indicate that CENP-A^{Cnp1} plays a direct role in recruiting the Ino80 complex to the centromeres. To test this hypothesis, we performed ChIP-Seq experiments for Ino80 subunits (Ies6-5xFlag and Iec1-5xFlag) in wild-type and CENP-A^{Cnp1} mutant (*cnp1-1*) cells grown at the restrictive temperature for *cnp1-1* (36°C for 3 hours) (**Fig. 3a**). Our ChIP-Seq analysis showed that the bindings of Iec1 and Ies6 over the central domain are diminished in *cnp1-1* cells and become constrained to more specific regions of the central domain, including the tRNA genes (**Fig. 3a**). These results indicate that the widespread associations of the Ino80 complex with CENP-A^{Cnp1} chromatin domain occur through CENP-A^{Cnp1}, although DNA sequence features may also play some role. We have included and discussed this in the **Results** (p10) and the **Discussion** (p13) sections. The results obtained from our neocentromere experiment (**Fig. 3c, d**) further suggest that CENP-A^{Cnp1} chromatin, not the DNA sequence, majorly contributes to localizing the Ino80 complex to the active centromere (p10, 13).

Specific Comment:

The paper would be improved for a general audience by additional introduction and discussion on what is already known about the specific functions of the Ino80 complex.

Our Response:

According to the reviewer's suggestion, we have added fully separated **Introduction** and **Discussion** sections to the revised manuscript. This also helped us integrate and discuss the previous literature that we overlooked in the prior version of the manuscript. In doing so, we added 23 new publications to the **References** section.

Specific Comment:

Related to Fig. S-1: SWR1 has a similar growth pattern to the hrp1Δ and ies2Δ. Does it have a similar decrease in CENP-A as seen in Fig. 1B/C with other mutants?

Our Response:

We would like to note that **Supplementary Figure 1b** shows that *swr1*Δ cells grow more slowly than *hrp1*Δ and *ies2*Δ cells, even on the non-selective (NS) media. Thus, we think that the reduced growth of *swr1*Δ cells on the FOA-containing media (+FOA) merely reflects the slow growth phenotype rather than defective silencing. Nevertheless, to address the reviewer's concern more unambiguously, we performed CENP-A^{Cnp1} ChIP analysis in wild-type and *swr1*Δ cells. As shown below, we found no significant reduction of CENP-A^{Cnp1} enrichment at the *cnt* region in *swr1*Δ cells.

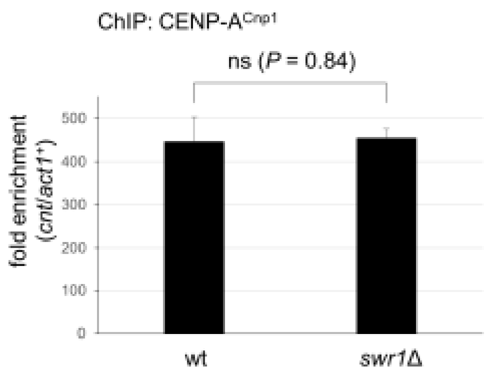

Specific Comment:

Related to Fig1: B: Please indicate the y-axis.

Our Response:

We modified **Figure 1b** to include CENP-A^{Cnp1} ChIP data obtained from other mutants of the Ino80 complex (*ies2*Δ, *iec3*Δ, *arp8*Δ and *iec1*Δ cells), in addition to *ies6*Δ. In this modified figure, we have properly labeled the y-axis as fold enrichment (*cnt*/*act1*$^{+}$).

Specific Comment:

Related to Fig. S 4: does ies2/rsc12Δ have similar growth as ies2Δ, or does it further elevate 5-FOA sensitivity?

Our Response:

To address the reviewer's question, we performed a *cnt1*:*ura4*$^{+}$ silencing assay with *rsc1*Δ *ies2*Δ cells. We found that this double mutation did not cause any additive silencing defect (**Supplementary Fig. 10d**). We have included this in the **Results** section (p9).

Reviewer #3

Specific Comment:

The possibility of indirect effects on centromere caused by mutations in Ino80 genes is not investigated. For example it is possible that Ino80 is required for expression of genes encoding CENP-A loading factors. This possibility should be ruled out by staying genome-wide gene expression changes in the mutants.

Our Response:

As suggested by the reviewer, we compared the gene expression profiles of *ies6*Δ and wild-type cells using mRNA-Seq analysis to address the issues of indirect effects. Consistent with

the notion that the Ino80 complex plays a role in transcription, we identified hundreds of genes whose mRNA levels are significantly down- or up-regulated in *ies6*Δ cells (**Supplementary Table 1**). However, among the genes whose functions are known to be important for CENP-A^{Cnp1} chromatin assembly, only *cnp3*$^{+}$, which encodes the *S. pombe* ortholog of CENP-A^{Cnp1}-binding protein, CENP-C^{Cnp3}, showed significantly reduced expression in *ies6*Δ cells (**Supplementary Table 1**). Notably, *ies6*Δ did not down-regulate *cnp3*$^{+}$ under a suboptimal growth condition (at 25°C in minimal EMM) (**Supplementary Fig. 9a**), but the *ies6*Δ cells still showed a significantly reduced level of CENP-A^{Cnp1} at the centromere under this condition (**Supplementary Fig. 9b**). Therefore, we conclude that the Ino80 complex regulates CENP-A^{Cnp1} chromatin regardless of its involvement in the regulation of *cnp3*$^{+}$ expression. We have included this in the **Results** section (p8, 9).

Specific Comment:

The silencing defects of iec1, ies2, iec3, arp8 and ies6 mutant strains is are not so clear from the spotting assays (Fig 1a). There is only a slight reduction of growth on FOA plates. I suggest performing an RT-PCR analysis of the cnt1:ura4 reporter mRNA to strengthen this result.

Our Response:

We agree with the reviewer that the silencing defects of Ino80 mutant (*iec1*Δ, *ies2*Δ, *iec3*Δ, *arp8*Δ and *ies6*Δ) cells are not robust, and that we need alternative assays to confirm that the integrity of CENP-A^{Cnp1} chromatin is defective in the mutant cells. Instead of performing RT-PCR analysis, which would improve only the indirect silencing assay, we decided to directly monitor CENP-A^{Cnp1} chromatin at *cnt1*:*ura4*$^{+}$ and the endogenous *cnt* region in wild-type and mutant cells (*iec1*Δ, *ies2*Δ, *iec3*Δ, *arp8*Δ, and *ies6*Δ) by performing ChIP analysis of CENP-A^{Cnp1}. We found that mutations in the Ino80 complex, most notably *iec1*Δ and *ies6*Δ, reduced the enrichments of CENP-A^{Cnp1} at *cnt1*:*ura4*$^{+}$ and the endogenous central domain (*cnt*) (**Supplementary Fig. 1c** and **Fig. 1b**). This new analysis strengthens our conclusion that the function of the Ino80 complex is important for CENP-A^{Cnp1} chromatin assembly. We have included this in the **Results** section (p5).

Specific Comment:

TBZ sensitivity and chromosome loss measurements (Fig 1d) should be carried out for all iec1, ies2, iec3, arp8 and ies6.

Our Response:

According to the reviewer's suggestion, we performed TBZ sensitivity assays and minichromosome loss assays in various Ino80-mutant cells (*iec1*Δ, *ies2*Δ, *iec3*Δ, *arp8*Δ, and *ies6*Δ). Consistent with the results of our CENP-A^{Cnp1} ChIP analysis, *iec1*Δ and *ies6*Δ (and also *arp8*Δ to a lesser extent) caused TBZ sensitivity and increased minichromosome loss (**Fig. 1d, e**). This analysis confirms that the impaired CENP-A^{Cnp1} chromatin assembly in mutants of the Ino80 complex disrupts normal centromere function. We have included this in the **Results** section (p5).

Specific Comment:

The ChIP-seq analysis of H3 is not suitable for global quantification. It needs to be performed using DNA spiking controls adding the same amount to each sample to control for global changes, and also the results should be validated by Q-PCR at several loci. There are two problems with the analysis (described in the manuscript on pages 11-12). First the authors have used same amount of immunoprecipitated (IP) DNA from each sample for the library preparation and secondly the total number of reads have been used for normalization of each CHIP-seq sample. A global increase of H3 would lead to an increase of the IP material yielding more IP DNA and more sequence reads in total but this cannot be detected by the current analysis. Therefore the conclusions made on page 5 about global effects of ies6 and hrp1 on H3 density are not valid.

Our Response:

According to the reviewer's suggestion, we decided to improve the histone H3 ChIP-Seq data by performing and analyzing quantitative H3 ChIP-Seq experiments that included

external spike-in controls (described in the **ChIP-Seq and data analysis** subsection of the **Methods** section). Compared to the previous H3 ChIP-Seq experiments, these new H3 ChIP-Seq analyses (**Fig. 2, Supplementary Fig. 3 and Supplementary Fig. 7**) showed far more clearly that *ies6*Δ causes both a prominent increase in histone H3 occupancy at centromeres and a global increase at genic regions. Our quantitative ChIP analysis confirmed that H3 levels are significantly increased at the centromere in *ies6*Δ cells (**Supplementary Fig. 8a, right panel**). Compared to the Ino80 complex, we found that $Chd1^{Hrp1}$ promotes histone H3 removal at similar non-centromeric genic regions (TSSs and TESs), but plays a much weaker role in evicting H3 from the euchromatin (**Fig. 2e and Supplementary Fig. 7a, b**). We have included and discussed these findings in the **Results** (p7) and **Discussion** (p14) sections.

Specific Comment:

The data shown in Supplementary Fig 3 are not conclusive since the double mutant strain is growing so slowly. A much better experiment is to test the H2AZ levels at centromeres in ies2 cells by ChIP.

Our Response:

As suggested by the reviewer, we performed ChIP analysis of $H2A.Z^{Pht1}$ in wild-type and *ies6*Δ cells. We found that, in sharp contrast to its ability to significantly increase H3 occupancy, *ies6*Δ does not cause a significant increase in $H2A.Z^{Pht1}$ occupancy at the central core region (**Supplementary Fig. 8a**). We also performed quantitative ChIP-Seq experiments for $H2A.Z^{Pht1}$ with the inclusion of external spike-in controls. The results of these experiments confirmed that the accumulation of $H2A.Z^{Pht1}$ at the centromeres in *ies6*Δ cells is marginal compared to the robust increase in H3 levels (**Supplementary Fig. 8b**). However, similar to what was shown in *S. cerevisiae*, we observed that the Ino80 complex plays a prominent role in removing histone $H2A.Z^{Pht1}$ from genic regions (**Supplementary Fig. 8b, d, e**). In the silencing assay intended to test whether elimination of $H2A.Z^{Pht1}$ could rescue the defective central core silencing observed in Ino80-mutant cells, we used *ies2*Δ cells instead of *ies6*Δ cells for the Ino80 mutant because we were unable to generate the *ies6*Δ *pht1*Δ double-mutant strain (which is likely to be synthetically lethal). The new ChIP-Seq analyses,

together with the silencing assay, strengthen our conclusion that the Ino80 complex regulates centromeric chromatin assembly in a manner independent of its role in restricting H2A.Z^{Pht1} localization. We have included and discussed these results in the **Results** (p7, 8) and **Discussion** (p14) sections.

Specific Comment:

A reference to Buchanan et al 2009 should be added along with reference 17 on page 4 line 12.

Our Response:

According to the reviewer's suggestion, we have cited the Buchanan et al. study in the revised manuscript (p7).

Again, we thank the reviewers for their time and effort reviewing our manuscript. The comments were constructive and, in addressing them, we have greatly improved our insight into the role of the Ino80 complex in centromeric chromatin assembly.

Sincerely,

Daeyoup Lee, PhD.

P.S. The following is a list of the major changes made to the revised manuscript:

1) To respond to a specific comment of Reviewer 2, we have fully separated the **Introduction** and **Discussion** sections in the revised manuscript. We also now include substantially more references (23 new references were added to the revised manuscript).

2) To respond to the general comments of Reviewers 1 and 2 regarding our citation of

the previous literature, we now cite and discuss the noted papers (Chambers et al., Ogiwara et al. and Hildebrand et al.) in the **Introduction** (p3, 4) and **Discussion** (p13, 14) sections.

3) To respond to general and specific comments of Reviewers 1, 2, and 3, we performed new histone H3 ChIP-Seq experiments that included external spike-in controls. The new ChIP-Seq analysis clearly shows prominent increases of H3 occupancies at the central domains, TSSs, and TESs in *ies6*Δ cells (**Fig. 2, Supplementary Fig. 3, and Supplementary Fig. 7**).

4) To respond to general and specific comments of Reviewers 1, 2, and 3, we performed conventional H2A.Z^{Pht1} ChIP and quantitative H2A.Z^{Pht1} ChIP-Seq analyses with external spike-in controls. The analyses showed that *ies6*Δ does not cause a significant increase in H2A.Z^{Pht1} occupancy at the centromere, which is in a sharp contrast to the significant increase in H3 occupancy seen in *ies6*Δ cells (**Supplementary Fig. 8a**). This strengthens our conclusion that the Ino80 complex affects CENP-A^{Cnp1} chromatin in a manner independent of its role in restricting H2A.Z^{Pht1} localization (p7, 8, 14).

5) To respond to specific comments of Reviewer 1, we performed ChIP-Seq experiments for Ino80 subunits (Ies6-5xFlag and Iec1-5xFlag) in wild-type and CENP-A^{Cnp1} mutant (*cnp1-1*) cells (**Fig. 3a**). Our results showed that the widespread association of the Ino80 complex with CENP-A^{Cnp1}-enriched domains requires CENP-A^{Cnp1}. This suggests that CENP-A^{Cnp1} may play a direct role in recruiting the Ino80 complex to centromeres, and provides the functional context in which the physical interaction of CENP-A^{Cnp1} with the Ino80 complex can have a biological meaning (p10, 13).

6) To respond to specific comments of Reviewers 2 and 3, we performed ChIP analysis of CENP-A^{Cnp1} in wild-type and Ino80 complex mutant (*iec1*Δ, *ies2*Δ, *iec3*Δ, *arp8*Δ, and *ies6*Δ) cells (**Supplementary Fig. 1c** and **Fig. 1b**). In addition, to respond to a specific comment of Reviewer 3, we repeated our TBZ sensitivity and minichromosome loss assays with all of the Ino80 mutants (*iec1*Δ, *ies2*Δ, *iec3*Δ, *arp8*Δ, and *ies6*Δ) used for the silencing assay. Together, the results of these analyses showed that the function of the Ino80 complex is critical for both centromere function and CENP-A^{Cnp1} chromatin assembly (p5).

7) To respond to general and specific comments of Reviewers 2 and 3, we used mRNA-

Seq analysis to compare the gene expression profiles of *ies6*Δ and wild-type cells. This analysis revealed that *cnp3*$^+$, which encodes the *S. pombe* ortholog of the CENP-A^{Cnp1}-binding protein, CENP-C^{Cnp3}, shows significantly reduced expression in *ies6*Δ cells (**Supplementary Table 1**). We addressed this issue by demonstrating that *ies6*Δ cells show significantly reduced CENP-A^{Cnp1} occupancy at the centromere even under a suboptimal growth condition, which allows *cnp3*$^+$ expression to be unaffected by *ies6*Δ (**Supplementary Fig. 9a, b**) (p8, 9).

8) To respond to a specific comment of Reviewer 1, we compared our results with those reported by Yen and Pugh, and discussed the similarities of the *S. pombe* and *S. cerevisiae* Ino80 complexes in their localizations and abilities to evict nucleosomes near the TSS (p7, 14).

9) To respond to a specific comment of Reviewer 1, we analyzed CENP-A^{Cnp1} accumulation at genic regions in *ies6*Δ cells in an effort to determine if the role of the Ino80 complex in the ectopic deposition of CENP-A^{Cnp1} in *S. cerevisiae* (Hildebrand et al.) is conserved in *S. pombe* (**Supplementary Fig. 7c**). We found that CENP-A^{Cnp1} accumulation does not occur at gene promoters where the Ino80 complex acts to remove H3-containing nucleosomes. We propose that this could reflect a divergence of Ino80 function in *S. cerevisiae*, and that this might have occurred during the evolution from regional to point centromeres (p13, 14).

10) To respond to a specific comment of Reviewer 2, we performed ChIP analysis of Scm3-GFP in wild-type and *ies6*Δ cells. We found that the binding of Scm3 to the central core region (*cnt*) is not significantly impaired in *ies6*Δ cells (**Supplementary Fig. 5**). This result and several of our other findings (**Fig. 2a**, and **Supplementary Fig. 4**) indicate that the CENP-A^{Cnp1} deposition pathway is likely to be intact in *ies6*Δ cells (p6, 7).

11) To respond to a specific comment of Reviewer 2, we performed a *cnt1*:*ura4*$^+$ silencing assay with *rsc1*Δ *ies2*Δ cells. We found that there was no additive silencing defect in the double mutant compared to either single mutant (**Supplementary Fig. 10d**) (p9).

12) To emphasize the main conclusion of our study, we moved the schematic model for the replacement of histone H3 with CENP-A^{Cnp1} at centromeres (previously **Supplementary Fig. 10**) to the main figures (**Fig. 5**) of the revised manuscript.

13) To respond to a specific comment of Reviewer 2, we modified **Figure 1b** such that the data presented for our CENP-A^{Cnp1} ChIP analysis include more mutants of the Ino80 complex (*ies2*Δ, *iec3*Δ, *arp8*Δ, *iec1*Δ, and *ies6*Δ). We also addressed the reviewer's comment by properly labeling the y-axis as fold enrichment (*cnt*/*act1*$^{+}$) in this modified figure.

14) To respond to a specific comment of Reviewer 2, we performed CENP-A^{Cnp1} ChIP analyses in wild-type and *swr1*Δ cells. As shown in the point-by-point responses to Reviewer 2, we found no significant reduction of CENP-A^{Cnp1} enrichment at the central core region (*cnt*) in *swr1*Δ cells. We decided not to include this result in the revised manuscript, as **Supplementary Figure 1b** shows that *swr1*Δ cells grow more slowly than *hrp1*Δ and *ies2*Δ cells even on non-selective (NS) medium. Thus, the reduced growth of *swr1*Δ cells on the FOA-containing media (+FOA) is likely to reflect this slow growth rather than a defective silencing phenotype.

15) To respond to a specific comment of Reviewer 3, we cited the Buchanan et al. study in the revised manuscript (p7).

16) To visualize the co-localization of Ies6 and Iec1 at genic regions (preferentially at the TSS and TES), we performed a heat map analysis for Ies6 and Iec1 sorted for the enrichment of Ies6 (**Supplementary Fig. 6b**). We also visualized the correlation between Ies6 binding and the increased H3 occupancy at genic regions of *ies6*Δ cells using heat maps for Ies6 binding and H3 occupancy changes in *ies6*Δ cells, both of which were sorted for the enrichment of Ies6 binding (**Fig. 2b**).

17) To detail the methods used to acquire and analyze the data from the newly added ChIP-Seq experiments that included external spike-in controls, we modified the **ChIP-Seq and data analysis** subsection of the **Methods** section (p18).

Reviewers' comments:

Reviewer #2 (Remarks to the Author):

The authors have added many new experiments to address the concerns raised in the initial round of review. In general, the paper is stronger in its conclusions. The authors did not add the controls and/or mutagenesis that would be required to make a conclusion about the directness of interaction between Ino80 and CENP-A (for Fig. 3b). Nonetheless there are multiple instances in the text, including in the results and the discussion sections that there is a 'physical interaction' and that it is direct. Figure 3b is just not strong data by itself; there is no compelling evidence of a direct/physical interaction and certainly the over-interpretation of these data needs to be pulled way back in the text. Also, they use the finding that many known centromeric chromatin factors weren't on their gene expression change list, except CENP-C, as support for their idea that the effect from Ino80 mutants reveals a direct role at centromeres. It wasn't clear from this table if there were actually data for the other centromere components and how one would assess how solid the conclusions could be made on the expression of those components from that experiment. I also am unconvinced that the new model figure (Fig. 5) is helpful; it seems to suggest communication between kinetochore proteins and Ino80, but such a connection is never addressed in the paper.

Reviewer #3 (Remarks to the Author):

The manuscript has been very much improved. However, I have some remaining points of concern that should be adressed before acceptance

1. It is clear that Ino80 plays an important role in H3 eviction and CENP-A deposition at centromeres together with the previously described remodeling activity by Hrp1. However by reading the abstract one gets the impression that Ino80 is the only relevant remodeling activity at centromeres.
The authors conclude on page 9 line 194
'These data indicate that Ino80 and Chd1Hrp1 share a role in promoting the replacement of histone H3 with CENP-ACnp1.'
This conclusion should also be mentioned in the abstract.

2. On page 7 lines 144-146
'We found that Chd1Hrp1 also promotes histone H3 removal at non-centromeric regions similar to those targeted by the Ino80 complex (near the start and end of genes), but it plays a much weaker role in evicting H3 from euchromatin (Fig. 2e and Supplementary Fig. 7a, b).'

According to Fig 2e and Supplementary Fig. 7b the effect of ies6 on H3 in euchromatin is about 2 fold stronger than hrp1. Thus, a much weaker effect is clearly an overstatement and should be changed to '…it plays a weaker role in evicting H3…'

3. In the introduction on page 4 lines 75-76
'In these studies, however, RSF1 and CHD1 were found to be only marginally or moderately involved in the deposition of CENP-A at the centromeres.'

Reference 21 is not properly cited in this context. It is unfair and unnecessary to describe the effect of CHD1 (Hrp1) as marginal.

Reviewer #2 (Remarks to the Author):

The authors have added many new experiments to address the concerns raised in the initial round of review. In general, the paper is stronger in its conclusions.

Our response: Thank you.

The authors did not add the controls and/or mutagenesis that would be required to make a conclusion about the directness of interaction between Ino80 and CENP-A (for Fig. 3b). Nonetheless there are multiple instances in the text, including in the results and the discussion sections that there is a 'physical interaction' and that it is direct. Figure 3b is just not strong data by itself; there is no compelling evidence of a direct/physical interaction and certainly the over-interpretation of these data needs to be pulled way back in the text.

Our response: According to the reviewer's suggestion, we modified figures and main text to avoid over-interpretation of the Co-IP result. First, we relocated the Co-IP data from main figures to supplementary figures (from Fig. 3b to Supplementary Fig. 12).Then, we modified the interpretation of the Co-IP data in the **Results** section in a more subtle way as below:

"We observed that CENP-A^{Cnp1}, when overexpressed, co-immunoprecipitates with Ies6, which raises the possibility that CENP-A^{Cnp1} may have an interaction with the Ino80 complex (Supplementary Fig. 12)" (p10).

Finally, we removed the sentence "The ability of CENP-A^{Cnp1} to physically interact with the Ino80 complex indicates that CENP-A^{Cnp1} plays a direct role in either recruiting the Ino80 complex or stably maintaining it at the centromeres" from the **Discussion** section (p13). We accordingly modified the paragraph including this sentence.

Also, they use the finding that many known centromeric chromatin factors weren't on their gene expression change list, except CENP-C, as support for their idea that the effect from Ino80 mutants reveals a direct role at centromeres. It wasn't clear from this table if there were actually data for the other centromere components and how one would assess how solid the conclusions could be made on the expression of those components from that experiment.

Our response: According to the reviewer's suggestion, we added the actual data for the gene expression changes of CENP-A^{Cnp1} chromatin-related genes in *ies6*Δ cells in Supplementary Table 1.

I also am unconvinced that the new model figure (Fig. 5) is helpful; it seems to suggest communication between kinetochore proteins and Ino80, but such a connection is never addressed in the paper.

Our response: According to the reviewer's suggestion, we removed the model figure (Fig. 5) and the associated sentence "At fission-yeast centromeres, the Ino80 complex may act in concert with the machineries of CENP-A^{Cnp1} deposition, such as Scm3, to mediate CENP-A^{Cnp1} chromatin assembly" from the **Discussion** section (p14).

Reviewer #3 (Remarks to the Author)

The manuscript has been very much improved.

Our response: Thank you.

However, I have some remaining points of concern that should be adressed before acceptance

1. It is clear that Ino80 plays an important role in H3 eviction and CENP-A deposition at centromeres together with the previously described remodeling activity by Hrp1. However by reading the abstract one gets the impression that Ino80 is the only relevant remodeling activity at centromeres.

The authors conclude on page 9 line 194

'These data indicate that Ino80 and Chd1Hrp1 share a role in promoting the replacement of histone H3 with CENP-ACnp1.'

This conclusion should also be mentioned in the abstract.

Our response: According to the reviewer's suggestion, we explicitly described the functional redundancy between the Ino80 complex and $Chd1^{Hrp1}$ in the **Abstract** (p2) as well as in the **Introduction** (p4) sections.

2. On page 7 lines 144-146

'We found that Chd1Hrp1 also promotes histone H3 removal at non-centromeric regions similar to those targeted by the Ino80 complex (near the start and end of genes), but it plays a much weaker role in evicting H3 from euchromatin (Fig. 2e and Supplementary Fig. 7a, b).'

According to Fig 2e and Supplementary Fig. 7b the effect of ies6 on H3 in euchromatin is about 2 fold stronger than hrp1. Thus, a much weaker effect is clearly an overstatement and should be changed to '...it plays a weaker role in evicting H3...'

Our response: According to the reviewer's suggestion, we modified the sentence as "but it plays a weaker role in evicting H3 from euchromatin… " (p7).

3. In the introduction on page 4 lines 75-76

'In these studies, however, RSF1 and CHD1 were found to be only marginally or moderately involved in the deposition of CENP-A at the centromeres.'

Reference 21 is not properly cited in this context. It is unfair and unnecessary to describe the effect of CHD1 (Hrp1) as marginal.

Our response: According to the reviewer's suggestion, we modified the sentence as "however, RSF1 and CHD1 were found to be only moderately involved in the deposition of CENP-A at the centromeres" (p4).

In addition to these changes, we added **mRNA-Seq analysis** subsection in the **Methods** section which was missing in the previous version of the manuscript, and in doing so, added a new reference (#53). We also removed unessential descriptions of the figure colors in the **Figure legends** section to make the legends more succinct. We highlighted all the changes in the main text.

We edited our manuscript appropriately to comply with Nature Communications' policies and format requirements, especially for the restrictions on the length of Methods' subheadings and the colors within the main and the supplementary figures. We also added a new paragraph which contains a brief summary of both the results and the conclusions in the end of the **Introduction** section. Deposition of sequencing data were done (GSE99589) and described in the **Data availability** subsection of the **Methods** section.

REVIEWERS' COMMENTS:

Reviewer #2 (Remarks to the Author):

The authors responded to my earlier criticisms

Reviewer #3 (Remarks to the Author):

The authors have still not properly responded to my previous point about the roles of Ino80 and Hrp1. As judged by the data presented in Figure 1c (CENP-A ChIP) and Figure 2a (H3 ChIP) the two remodelling factors have similar effects on reduced CENP-A and increased H3 levels at centromeres.

Therefore conclusion on page 4 should be changed
'We find that the Ino80 complex plays a major role in CENP-ACnp1 chromatin assembly at the fission yeast centromeres in a redundant manner with Chd1Hrp1 which plays a minor role, and the role of the Ino80 complex is linked to removal of histone H3-containing nucleosomes.'

I disagree that there is a major and minor role. Instead the two different remodelling activities act together at centromeres with roughly equal importance.

Response to reviewer's comments (NCOMMS-16-27196B)

We revised our manuscript to answer the questions of reviewer #3. Details of the changes are described below.

Reviewer #2 (Remarks to the Author):

The authors have still not properly responded to my previous point about the roles of Ino80 and Hrp1. As judged by the data presented in Figure 1c (CENP-A ChIP) and Figure 2a (H3 ChIP) the two remodelling factors have similar effects on reduced CENP-A and increased H3 levels at centromeres.

Therefore conclusion on page 4 should be changed 'We find that the Ino80 complex plays a major role in CENP-A^{Cnp1} chromatin assembly at the fission yeast centromeres in a redundant manner with Chd1Hrp1 which plays a minor role, and the role of the Ino80 complex is linked to removal of histone H3-containing nucleosomes.'

I disagree that there is a major and minor role. Instead the two different remodelling activities act together at centromeres with roughly equal importance.

Our response: According to the reviewer's comment, we have modified the conclusion on page 4 as below.

"We find that the Ino80 complex plays an important role in CENP-A^{Cnp1} chromatin assembly at the fission yeast centromeres in a redundant manner with Chd1^{Hrp1}, and the role of the Ino80 complex is linked to removal of histone H3-containing nucleosomes. Consistent with a direct role in CENP-A^{Cnp1} chromatin assembly, the Ino80 complex is associated with centromeric regions in a CENP-A^{Cnp1}-dependent manner and when tethered to a non-centromeric DNA inserted in an endogenous centromere promotes spreading of CENP-A^{Cnp1} chromatin into the non-centromeric DNA. Thus, CENP-A^{Cnp1} chromatin utilizes the Ino80 complex together with Chd1^{Hrp1} to mediate replacement of histone H3 with CENP-A^{Cnp1} at regional centromeres of fission yeast."

In addition to this, we have toned down the conclusions throughout the manuscript in such a way that the two chromatin remodelling factors (the Ino80 complex and Chd1^{Hrp1}) act together at centromeres to mediate replacement of histone H3 with CENP-A^{Cnp1} (please see the track changes in p2, p4, p5 and p14).

We appreciate your time and efforts in reviewing this manuscript.
